# C3G forms complexes with Bcr-Abl and p38α MAPK at the focal adhesions in chronic myeloid leukemia cells: implication in the regulation of leukemic cell adhesion

**DOI:** 10.1186/1478-811X-11-9

**Published:** 2013-01-23

**Authors:** Vera Maia, Sara Ortiz-Rivero, María Sanz, Javier Gutierrez-Berzal, Indira Álvarez-Fernández, Sara Gutierrez-Herrero, Jose María de Pereda, Almudena Porras, Carmen Guerrero

**Affiliations:** 1 Centro de Investigación del Cáncer, IBMCC, CSIC-Universidad de Salamanca, Salamanca, Spain; 2 Departamento de Bioquímica y Biología Molecular II, Facultad de Farmacia, UCM, Instituto de Investigación Sanitaria del Hospital Clínico San Carlos (IdISSC), Madrid, Spain; 3 Departamento de Medicina, Facultad de Medicina, Universidad de Salamanca; Instituto de Investigación Biomédica de Salamanca (IBSAL), Salamanca, Spain

**Keywords:** C3G, p38α MAPK, Cbl, p130Cas, Abi1, Bcr-Abl, CML, Cell adhesion

## Abstract

**Background:**

Previous studies by our group and others have shown that C3G interacts with Bcr-Abl through its SH3-b domain.

**Results:**

In this work we show that C3G and Bcr-Abl form complexes with the focal adhesion (FA) proteins CrkL, p130Cas, Cbl and Abi1 through SH3/SH3-b interactions. The association between C3G and Bcr-Abl decreased upon Abi1 or p130Cas knock-down in K562 cells, which suggests that Abi1 and p130Cas are essential partners in this interaction. On the other hand, C3G, Abi1 or Cbl knock-down impaired adhesion to fibronectin, while p130Cas silencing enhanced it. C3G, Cbl and p130Cas-SH3-b domains interact directly with common proteins involved in the regulation of cell adhesion and migration. Immunoprecipitation and immunofluorescence studies revealed that C3G form complexes with the FA proteins paxillin and FAK and their phosphorylated forms. Additionally, C3G, Abi1, Cbl and p130Cas regulate the expression and phosphorylation of paxillin and FAK. p38α MAPK also participates in the regulation of adhesion in chronic myeloid leukemia cells. It interacts with C3G, CrkL, FAK and paxillin and regulates the expression of paxillin, CrkL and α5 integrin, as well as paxillin phosphorylation. Moreover, double knock-down of C3G/p38α decreased adhesion to fibronectin, similarly to the single silencing of one of these genes, either C3G or p38α. These suggest that C3G and p38α MAPK are acting through a common pathway to regulate cell adhesion in K562 cells, as previously described for the regulation of apoptosis.

**Conclusions:**

Our results indicate that C3G-p38αMAPK pathway regulates K562 cell adhesion through the interaction with FA proteins and Bcr-Abl, modulating the formation of different protein complexes at FA.

## Background

C3G is a guanine nucleotide exchange factor (GEF) for Rap1 and R-Ras, two members of the Ras family of small GTPases. C3G is a 140 kDa protein, build up with several modular domains clearly differentiated, both structurally and functionally. These comprise a REM-CDC25-H domain, which contains the catalytic or “GEF” domain, and a large proline-rich domain or SH3-binding (SH3-b) domain that interacts directly with Crk isoforms and other SH3-containing proteins such as p130Cas, Hck and c-Abl [1-3]. C3G-mediated Rap1 activation plays critical roles in adhesion. In fact, C3G-Rap1 pathway is essential during early mouse embryogenesis, due to its role in integrin- and paxillin-mediated cellular adhesion and spreading [4]. Moreover, C3G is required for the formation and stabilization of β1-integrin and paxillin-positive FAs. C3G is also an essential activator of Rap1 during cell junction formation, both in epithelial and endothelial cells (revised by [5]). In addition, C3G has been implicated in Rap1-dependent adhesion in many hematopoietic-cell types [6,7].

We have previously identified a truncated C3G isoform, named p87C3G, which is abundantly expressed in chronic myeloid leukemia (CML) cells. p87C3G interacts with Bcr-Abl and is phosphorylated through a Bcr-Abl-dependent mechanism [8]. Pull-down and immunoprecipitation studies revealed that the interaction between p87C3G and Bcr-Abl involves the SH3-b domain (mainly the second poly-proline stretch) of p87C3G and the SH3 domain of Bcr-Abl. However, this interaction is not direct, as assessed by Two-Hybrid analysis (C. Guerrero, unpublished results). In addition, recent reports have described an interaction between C3G and c-Abl, which involves the C3G SH3-b domain, as demonstrated by *in vitro* experiments the involvement of the C3G SH3-b domain in this interaction [3,9]. The existence of an interaction between C3G and Bcr-Abl through CrkL has also been suggested, although this interaction would involve the SH3-b domain of Abl [10,11].

It is known that Bcr-Abl induces abnormalities in the cytoskeletal function and alters normal interactions between FA proteins and their targets, thus disturbing normal adhesion. Specifically, Bcr-Abl interacts with FA proteins, such as p130Cas, paxillin, tensin and FAK. Bcr-Abl induces p130Cas phosphorylation and its constitutive binding to CrkL, disrupting the normal interaction between p130Cas and tensin [12]. Additionally, Bcr-Abl is involved in the regulation of the leukemic cells adhesion to laminin, fibronectin and collagen through the complex formation with integrin α2β1, being the Abl-SH3 domain the responsible of these effects [13]. As a result, CML cells have a reduced capacity to adhere to stromal layers and to fibronectin but show increased adhesion to laminin and collagen type IV [14,15]. This is important since altered adhesion to extracellular matrix proteins could lead to premature release of CML cells from the bone marrow, resulting in a deregulated hematopoiesis.

Recently, we have described a functional relationship between C3G and p38α MAPK in the regulation of apoptosis in CML cells and in MEFs [16,17]. Another common issue is that, similarly to C3G, p38MAPKs play important roles in the regulation of cell adhesion and migration processes [18,19]. p38 MAPK is involved in the migration of mesoderm during the embryogenesis [20] and mediates migration of several cell types, including tumor cells [21]. p38 MAPK also regulates adhesion; cells lacking p38α showed increased adhesion to several ECM proteins [18,22], which correlates with increased phosphorylation of the FA proteins FAK and paxillin [18]. These results indicate that p38α negatively regulates cell adhesion.

The role of the adapter proteins CrkL, p130Cas and Cbl in CML is well documented [1,12,23,24], and the association between Cbl and C3G, through CrkL, has been described in CML cells, fibroblasts, NK cells and T-cells [11,25-28]. Direct interaction between C3G and p130Cas has also been reported [29]. Interestingly, all these proteins contain SH3 and/or SH3-b domains and participate in cellular adhesion processes, being potential mediators of the Bcr-Abl/C3G interaction.

On the other hand, several Abl SH3 binding proteins have been identified, such as 3BP-1 [30], Abi1 [31], Abi2 [32], AAP1 [33], RIN1 [34], and PAG [35]. Remarkably, Abi1/2 has both, SH3 and SH3-b domains, which would allow its simultaneous interaction with Bcr-Abl and C3G. This arises the possibility that Abi1/2 may act as a mediator in the C3G/Bcr-Abl interaction.

In this work we have investigated possible mediators of the C3G-SH3-b/Bcr-Abl-SH3 domains interaction. Considering that the SH3 domain of Abl is the one involved in the regulation of the leukemic cells adhesive and invasive properties, one of the hallmarks of the pathogenesis of CML [13], and knowing the role of C3G in cellular adhesion, we hypothesize that C3G could modulate CML cells adhesiveness through its interaction with Bcr-Abl at the FAs. We have also evaluated the participation of p38α MAPK in the regulation of adhesion in CML and its functional interaction with C3G.

## Results

### The Bcr-Abl SH3-domain interacts with C3G, Abi1, Cbl and p130Cas

**Figure 1:**
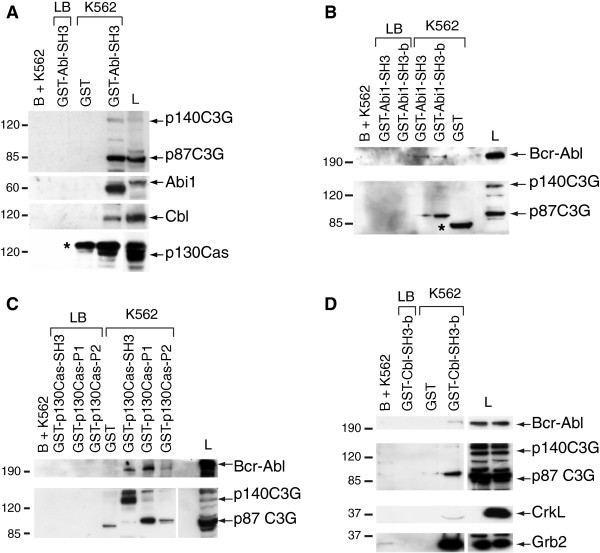
**Bcr-Abl and C3G interact with Cbl, Abi1 and p130Cas. (A)** Whole cell extracts from K562 were subjected to pull-down assays using GST-Abl-SH3 construct. The presence of C3G, Abi1, Cbl and p130Cas in the complexes was detected by inmunoblotting with specific antibodies. Representative Western blots of pull-down assays in K562 lysates using **(B)** Abi1-SH3 and SH3-b domains, **(C)** p130Cas-SH3, P1 and P2 domains, and **(D)** Cbl-SH3-b domain fused to GST as baits. The immunoblots were developed with specific antibodies against the indicated proteins. Glutathione-sepharose beads with whole cell lysate (B + K562), lysis buffer with the corresponding GST-fused proteins (LB) and pGEX-4T-1 empty plasmid with K562 whole cell lysate (GST) were used as negative controls. L: whole cell lysate (40 μg). The asterisks indicate unspecific bands.

Previous studies by our group and others have demonstrated the existence of functional interactions between, either C3G and/or its isoform p87C3G, and Bcr-Abl in K562 CML cells [8,10]. The interaction between p87C3G and Bcr-Abl involves the SH3 domain of Abl and the SH3-b domain of C3G [8]. However, Two-Hybrid analysis indicated that this binding was not direct (data not shown), suggesting the participation of intermediates in this interaction. Based on that, we tried to identify potential mediators in the Bcr-Abl/C3G interaction among a set of proteins involved in cell-ECM (extracellular matrix) adhesion, which also participate in signaling pathways involving C3G or Bcr-Abl. The putative candidates are CrkL, p130Cas, Cbl and Abi1/2. To characterize these interactions, we performed pull-down assays in K562 lysates using a GST-Abl-SH3 construct as bait [8]. Figure 1A shows that the SH3 domain of Abl interacts with Cbl, p130Cas and p140C3G, besides the known interaction with Abi1/2 and p87C3G [8,31,32].

### Abi1-SH3/SH3-b, Cbl-SH3-b and p130Cas-SH3/SH3-b domains interact with C3G and Bcr-Abl

To study these interactions in depth, we performed pull-down assays, in K562 lysates, using the SH3 and SH3-b domains of Abi1, Cbl and p130Cas fused to GST as baits. Both Abi1-SH3 and SH3-b domains bound to Bcr-Abl, in agreement with the literature [31]. In addition, both domains interacted with p87C3G and a slight interaction of the Abi1-SH3-b domain with p140C3G was also detected (Figure 1B). Regarding p130Cas, Figure 1C shows that the three tested domains (SH3-binding, P1 and P2) interact with Bcr-Abl, albeit with different affinities. Additionally, p130Cas-SH3 domain interacted with p140C3G, in agreement with other studies [29], while P1 and P2 domains associate preferentially with p87C3G. On the other hand, Cbl-SH3-b domain clearly interacts with both Bcr-Abl and p87C3G (Figure 1D). Association of Cbl with Bcr-Abl, as well as with CrkL or Grb2 (used as controls) in K562 cells has been described previously [24], although the interaction described by these authors involved the Bcr-Abl SH2 domain and Cbl phospho-tyrosines. The preferential interaction of p87C3G over p140C3G with most of the tested domains probably reflects its highest expression in CML cells (Additional file 1) [8].

**Figure 2:**
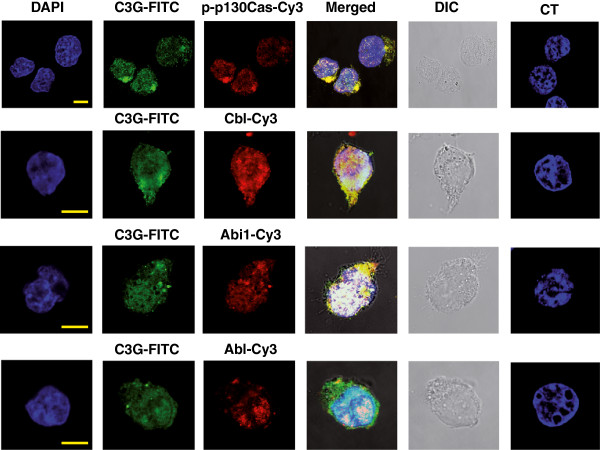
**C3G colocalizes with phospho-p130Cas, Cbl, Abi1 and Bcr-Abl in K562 cells.** Confocal microscopy images of K562 cells adhered to fibronectin covered slides (10 μg/ml) and labeled with anti-C3G (G4, mouse monoclonal, rows 1 and 2 or 1008, rabbit polyclonal, rows 3 and 4) and either, anti-phospho-p130Cas (rabbit polyclonal), anti-Cbl (rabbit polyclonal), anti-Abi1 (mouse monoclonal) or anti-Abl (mouse monoclonal) specific antibodies as indicated. Anti-FITC and anti-Cy3 were used as secondary antibodies. Nuclei were labeled with DAPI. Control cells (CT) were incubated with DAPI plus the secondary antibodies. DIC: Differential interference contrast microscopy. p-p130Cas: phospho-p130Cas. The bars represent 10 μm.

These experiments support the results derived from the Bcr-Abl-SH3 domain pull-down assays and suggest that all these proteins form complexes. In fact, all of them coimmunoprecipitate (Additional file 2). Therefore, Cbl, Abi1 and p130Cas are clear candidates to act as intermediates in the interaction between Bcr-Abl and C3G in K562 cells. In agreement with pull-down assays, confocal microscopy revealed a colocalization of C3G with phospho-p130Cas, Cbl, Abi1 and Bcr-Abl (Figure 2), which further supports that all these proteins form complexes in K562 cells.

### Interaction of Bcr-Abl with CrkL

CrkL, the major substrate of the tyrosine kinase Bcr-Abl, interacts directly with Bcr-Abl through its amino terminal SH3 domain and the SH3-binding region of Abl [36,37]. Here we show that the Abl-SH3 domain also interacts with CrkL (Additional file 3A). One possible explanation is that CrkL, similarly to CrkII, has a putative SH3-b domain inside the SH2 domain that could mediate this interaction [38]. Sequence alignment revealed that CrkL lacks the SH3-b domain present in CrkII, although it preserves a canonical SH3-b PXXP motif that itself is sufficient for binding to SH3 domains [29,36] (Additional file 3B). However, the CrkL-SH2 domain does not interact with Bcr-Abl, either by pull-down experiments (Additional file 3C) or in Two-Hybrid analysis (Additional file 4: Method 1 and Additional file 3D), indicating that the interaction between CrkL and Bcr-Abl, involving the Abl-SH3 domain, is not direct. An indirect interaction between these two proteins independent of the Bcr-Abl proline rich domain has been previously suggested [39].

### Abi1 and p130Cas knock-down alter the interaction between C3G and Bcr-Abl

**Figure 3:**
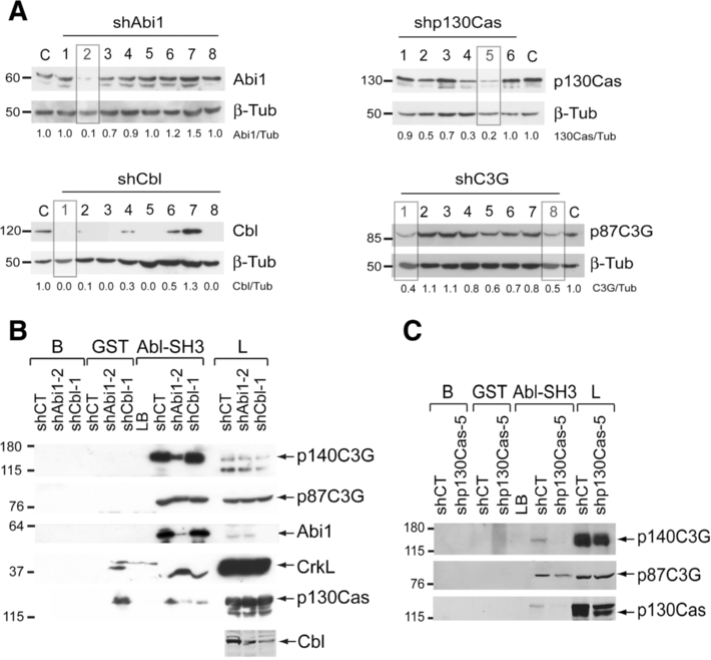
**Abi1 and p130Cas silencing alter Bcr-Abl/C3G interaction. (A)** Western blot analysis of the expression of Abi1, Cbl, p130Cas and p87C3G in whole cell lysates from K562 clones stably transfected with the indicated lentiviral shRNA particles. Relative Abi1/β-tubulin, Cbl/β-tubulin, p130Cas/β-tubulin and C3G/β-tubulin ratios are shown. shAbi1-2, shCbl-1, shp130Cas-5, shC3G-1 and shC3G-8 were selected as representative knock-down clones in the experiments. C: whole cell lysate of a K562 clone expressing control lentiviral shRNA. Pull-down assays with lysates of K562 shAbi1-2, shCbl-1 and shControl (shCT) clones **(B)** or K562 shp130Cas-5 and shCT clones **(C)**, using Abl-SH3 domain fused to GST as bait. p140C3G, p87C3G, Abi1, CrkL, Cbl and p130Cas expression was detected with specific antibodies. Panels showing p140C3G and p87C3G in Figure 3B correspond to different exposure times (longer for p140 and shorter for p87) for a better visualization of both isoforms. Beads with lysis buffer (B), GST construct with K562 whole cell lysate (GST) and K562 whole cell lysate with lysis buffer (LB) were used as negative controls. L: whole cell lysate (40 μg).

To determine the contribution of these proteins to the formation of C3G/Bcr-Abl complexes, we studied the interaction between C3G and Bcr-Abl in K562 clones upon silencing of Abi1, Cbl or p130Cas (Figure 3A). Figure 3B and 3C show that Abi1 and p130Cas knock-down clearly decreased Bcr-Abl/p140C3G interaction. p130Cas knock-down also decreased the interaction between Bcr-Abl and p87C3G that was unaffected by Abi1 knock-down. In contrast, Cbl silencing had no effect on these interactions (Figure 3B). On the other hand, the interaction between CrkL and Bcr-Abl increased in Abi1 silenced cells, suggesting that CrkL and Abi1 compete for their binding to Bcr-Abl (Figure 3B). Furthermore, both Abi1 and Cbl silencing decreased the interaction between Bcr-Abl and p130Cas. These results suggest that all these proteins interact with each other and form complexes in CML cells.

### C3G, Cbl and p130Cas bind directly to common adhesion proteins

To examine whether the interactions between the SH3 and/or SH3-b domains of the studied proteins were direct, we performed hybridizations with SH3 domain arrays (Panomics) containing 38 SH3 domains (Array I) and 36 SH3 domains (Array II) (Additional files 5, 6, 7 and Additional file 8: Table S1 and Additional file 9:Table S2), using His-tagged-SH3-b domains from C3G, Cbl and p130Cas (P2). The C3G-SH3-b domain directly associates with the SH3 domains of LCK, SPCN, cortactin, Yes1, Abl2 (ARG), SLK, c-Src, Hck, VAV-D2, Y124, PEXD, BTK, and Stam and with less affinity to NOF2-D1, VAV-D1, Abl and PLCγ (Additional file 5, Array I). Results with the array II confirmed the direct binding of C3G with c-Src and Abl2 and revealed its direct binding to NCK1-D2, OSF, Tec, PIG2, VINE-D3 and, with less affinity, PI3α. The known binding to CRKL-D1 was confirmed in this experiment [40] and contrarily to what we expected, no direct interaction with the Abi2-SH3 domain (AbI2B) was found. Binding to p130Cas-SH3 domain (BCA1) was not detected because the C3G-SH3-b fragment used lacks the upstream sequence involved in this interaction [29].

Similarly to the C3G-SH3-b domain, the Cbl-SH3-b domain clearly binds to LCK, SPCN, cortactin, Yes1, Abl2, SLK, c-Src, Hck, VAV2-D2, NOF2-D1, VAV-D1, Y124, PEXD, BTK, Stam and Abl (array I), Abl2, CrkL-D1, NCK1-D2, OSF, PI3α, Tec, PIG2, VINE-D3 and c-Src (array II) (Additional file 6). Interaction with PI3kα has been reported [24]. Also, in agreement with previous findings, a direct, although weak, interaction between the Cbl-SH3-b domain and the CrkL-SH3 (CrkL-D1) domain was observed [41], although in CML cells Cbl interacts preferentially with the CrkL-SH2 domain [24]. In contrast to C3G, Cbl also interacts directly with Itk, Dlg2, ITSN-D1 and TXK (array I) AbI2B (Abi-2), M3KA, SNX9, VAV3-D2, and SH3-1 (SH3-containing GRB2-like protein 1), being this last one in agreement with published data [42].

The p130Cas-P2 domain (SH3-b domain-2) renders a less specific hybridization, probably due to the smaller size of this fragment. Similarly to C3G and Cbl, it presents a clear direct association with LCK, Cortactin, Yes1, Abl2, SLK, c-Src, Hck, VAV2-D2, Y124, PEXD, BTK, ITSN-D1 (array I), Abl2, NCK1-D2, OSF, PI3α, Tec, PIG2, VINE-D3 and c-Src, (array II) and it binds with less affinity to Stam, BLK, Abl and CrkL-D1 (Additional file 7). Similarly to Cbl, p130Cas-P2 binds with high affinity to Itk, ITSN-D1 (array I), AbI2B, SH3-1 and SNX9 and weakly to M3KA and VAV3-D2. p130Cas-P2 presents an exclusive strong interaction with MLPK3, PSD95, PI3-ß (array I), GRB2L-D1, NE-DLG and NOF2-D1 (array II), and binds with less affinity to amphiphysin, RasGAP (array I), CSKP, BIN1 and EFS. The direct interaction between p130Cas-P2 and CrkL-SH3 domain (CRKL-D1) contrasts with previous reports showing a direct association between CrkL and p130Cas through the CrkL-SH2 domain in Bcr-Abl expressing cells and CML patients [12].

### C3G, Cbl, Abi1 and p38α MAPK knock-down expression inhibits K562 adhesion to fibronectin, while p130Cas silencing increases it

**Figure 4:**
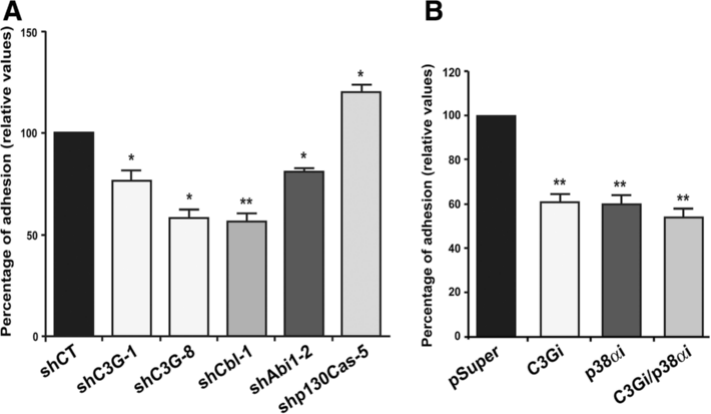
**C3G, Cbl, Abi1, p130Cas and p38α MAPK regulate adhesion to fibronectin in K562 cells. (A)** Percentage of adhesion to fibronectin of K562 cells upon knock-down of C3G (shC3G-1 and shC3G-8), Cbl (shCbl-1), Abi1 (Abi1-2) or p130Cas (shp130Cas-5). **(B)** Percentage of adhesion to fibronectin of K562 clones expressing interference RNAs (cloned in pSuper.gfp/neo) for C3G and/or p38α MAPK. The values are the mean ± SEM of at least three independent experiments. All values are relative to K562 cells transfected with control shRNA or empty pSuper.gfp/neo vector (pSuper). *p<0.05; ** p<0,01.

We next studied the ability of K562 clones with silenced expression of C3G, p38α MAPK, Cbl, Abi1 or p130Cas to adhere to fibronectin, which is the optimal adhesion substrate for K562 cells (Additional file 10), as compared to laminin or collagen IV. C3G, Cbl, Abi1 or p38α knock-down reduced the adhesion capacity of K562 cells while p130Cas silencing increased it (Figure 4A). Curiously, the double C3G and p38α MAPK knock-down did not additionally decreased adhesion as compared to each single knock-down (Figure 4B). This indicates that both proteins are likely acting through a common regulatory pathway to regulate adhesion in K562 cells.

### C3G and p38α MAPK form complexes with focal adhesion proteins

Cell adhesion to the extracellular matrix is mediated by integrins through regulation of the formation of different FA complexes, being a bidirectional cross-talk between integrins and FA proteins [43]. These complexes are constituted by protein kinases, such as FAK, and scaffold proteins, such as paxillin or p130Cas. In CML cells, it is known that paxillin interacts with protein kinases such as Src, p38 MAPK, c-Abl and FAK [44] and with Bcr-Abl through CrkL [45]. C3G also interacts with Bcr-Abl [8] and p130Cas [29], which agrees with data presented here.

**Figure 5:**
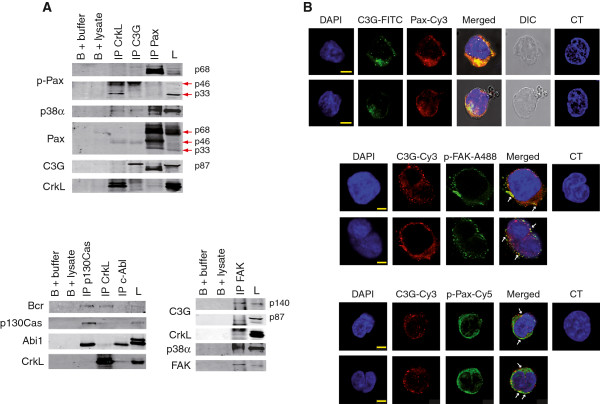
**C3G and p38α MAPK interact with FA proteins.** (**A)** Representative immunoprecipitation assays (IP), using the indicated antibodies, performed with protein extracts from K562 cells cultured on 10 μg/ml fibronectin for 24 h. Expression of p33, p46 and p68 phospho-paxillin (p-Pax) and paxillin isoforms, p38α, p87C3G, CrkL, Bcr-Abl, p130Cas, Abi1 and FAK was detected by immunoblotting with specific antibodies. GammaBind G Sepharose beads (B) with either buffer or whole cell lysate (lysate) were used as negative controls. **(B)** Confocal microscopy of K562 cells adhered for 24 h to slides covered with 10 μg/ml fibronectin and labeled with the indicated antibodies. Nuclei were labeled with DAPI. Control cells (CT) were incubated with DAPI plus the secondary antibodies. C3G-1008 (rabbit polyclonal) was used with Pax (mouse monoclonal) and p-FAK (goat polyclonal). C3G-G4 (mouse monoclonal) was used with p-Pax (rabbit polyclonal). C3G colocalizes with p-Pax and p-FAK in a punctuated pattern (white arrows). DIC: Differential interference contrast microscopy. Pax: paxillin. A488: Alexa Fluor 488. The bars represent 5 μm.

To characterize the interactions between some of these FA proteins and C3G or p38α MAPK in CML cells, we performed immunoprecipitation assays in whole cell lysates of K562 cells grown in the presence of fibronectin (10 μg/ml). Results from Figure 5A show that C3G interacts with FAK, paxillin, phospho-paxillin p68 isoform and p38α, apart from the already described interaction with CrkL (difficult to see probably due to the competition between C3G and Bcr-Abl for the binding to CrkL). Association of C3G with paxillin and FAK was also supported by immunofluorescence data (Figure 5B). Colocalization of C3G with p-Pax and p-FAK further supports a functional relationship between C3G and these focal adhesion proteins. p38α MAPK forms complexes with paxillin, as described before [44], C3G, FAK and CrkL. In agreement with previous studies, CrkL interacts with C3G, Bcr-Abl and phospho-paxillin p33 isoform [15,46-48] and Abi1 interacts with c-Abl [31,32]. The interactions C3G-p38α, CrkL-FAK, FAK-p38α, Abi1-p130Cas and CrkL-p38α have not been previously reported, although Crk can induce p38 MAPK phosphorylation in some cell types [49].

### C3G modulates the expression and activation of focal adhesion proteins

**Figure 6:**
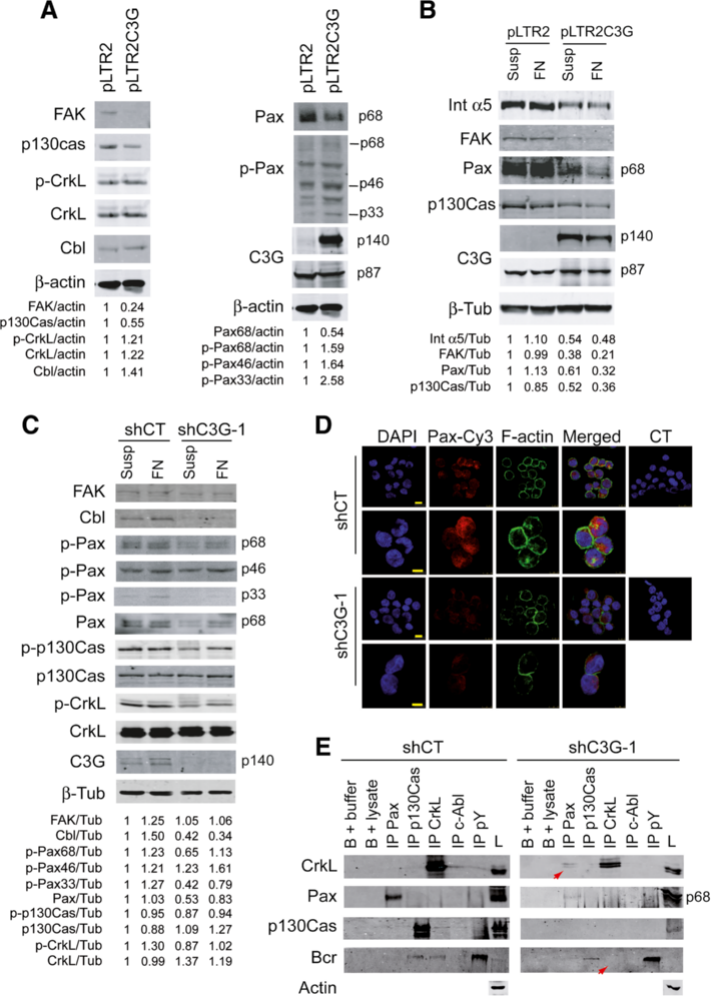
**C3G modulates the expression and activation of FA proteins. (A)** Western blot analysis of whole cell lysates (50 μg of protein) from K562 cells stably transfected with pLTR2C3G or the empty pLTR2 vector grown in suspension for 24 h. Paxillin (Pax), Cbl, CrkL, FAK, p130Cas and integrin α5 (Int α5) expression and paxillin and CrkL phosphorylation were detected with specific antibodies **(B)** Comparative analysis of the expression of Int α5, FAK, Pax and p130Cas in the above K562 clones grown with 10 μg/ml fibronectin (FN) for 24 h or in suspension (Susp). β-actin and β-tubulin (ß-Tub) levels were used as loading controls. **(C)** Western blot analysis of whole cell lysates from K562 cells stably transfected with shC3G (clone 1) or shCT lentivirus, either grown with fibronectin (FN) or in suspension (Susp) for 24 h. FAK, Cbl, paxillin, p130Cas and CrkL expression and paxillin, p130Cas and CrkL phosphorylation were detected. Relative ratios between the levels of these proteins and ß-tubulin are shown. **(D)** Paxillin expression is decreased in C3G silenced cells. Confocal microscopy of control (shCT) and shC3G-1 K562 cells adhered to fibronectin and labeled with anti-paxillin-Cy3 and anti-phalloidin antibodies. Nuclei were labeled with DAPI. Control cells (CT) were incubated with DAPI plus the secondary antibodies. The bars represent 10 μm (rows 1 and 3) and 7.5 μm (rows 2 and 4). **(E)** Immunoprecipitation assays (IP) of K562 cell lysates expressing, either shC3G or shCT, with the indicated antibodies followed by Western blot analysis of CrkL, Paxillin, p130Cas and Bcr expression. GammaBind G Sepharose beads (B) with either buffer or whole cell lysate (lysate) were used as negative controls. L: total cell lysate, Pax: paxillin, pY: phospho-tyrosine.

Next, we analyzed whether C3G overexpression affects the expression and/or phosphorylation of some FA proteins. Indeed, C3G overexpression led to a clear decrease in paxillin, FAK, p130Cas and integrin α5 protein levels. In contrast, an increase in phospho-paxillin isoforms, mainly p46 and p33, was also observed (Figure 6A). The decrease in the expression of FAK, p130Cas, integrin α5 and particularly paxillin was more evident in the presence of fibronectin (Figure 6B), indicating that an excess of C3G downregulates the outside-in signaling by a negative feed back mechanism. The increased levels of phospho-paxillin could be related with this regulation. Cbl protein levels slightly increased in C3G overexpressed cells, while CrkL expression and phosphorylation was not affected (Figure 6A).

To confirm these results through a complementary experimental approach, we analyzed the expression of these FA proteins in K562 C3G knock-down clones. Similarly to that observed in C3G overexpressed clones, paxillin expression decreased in C3G knock-down cells (Figure 6C). The decrease in paxillin expression was confirmed by immunofluorescence confocal microscopy of K562 cells attached to fibronectin (Figure 6D). In contrast, the levels of phospho-paxillin p33 and p68 isoforms decreased in C3G silenced clones, while they were increased by C3G overexpression, (Figure 6C). Additionally, a decrease in Cbl protein levels and a slight decrease in phospho-CrkL were also observed in the C3G knock-down clones, while no significant changes in p130Cas and CrkL levels were observed (Figure 6C). Fibronectin partially reversed the effect of C3G silencing on paxillin expression and phosphorylation, in contrast to the effect of fibronectin in C3G overexpressing clones. This is in agreement with the participation of C3G in the regulation of the outside-in pathway triggered by fibronectin.

We next analyzed the effect of C3G knock-down on FA proteins interactions by immunoprecipitation assays performed in control (shCT) and C3G knock-down K562 cells grown on fibronectin. Remarkably, C3G silencing produced a decrease in the association between CrkL and Bcr-Abl, while it promoted the CrkL-paxillin interaction (marked by arrows) characteristic of tumoral cells (Figure 6E).

Collectively, our data suggest that C3G plays a role in the regulation of the expression of FA proteins, and in their activation and association dynamics. Similarly to our previous published data on apoptosis, C3G seems to play a dual role in the regulation of cell adhesion, as both upregulation and downregulation of C3G expression have similar effect on the expression of proteins involved in the signaling pathways regulating cell adhesion.

### p38α MAPK regulates FA protein expression and activation in a C3G antagonistic fashion

**Figure 7:**
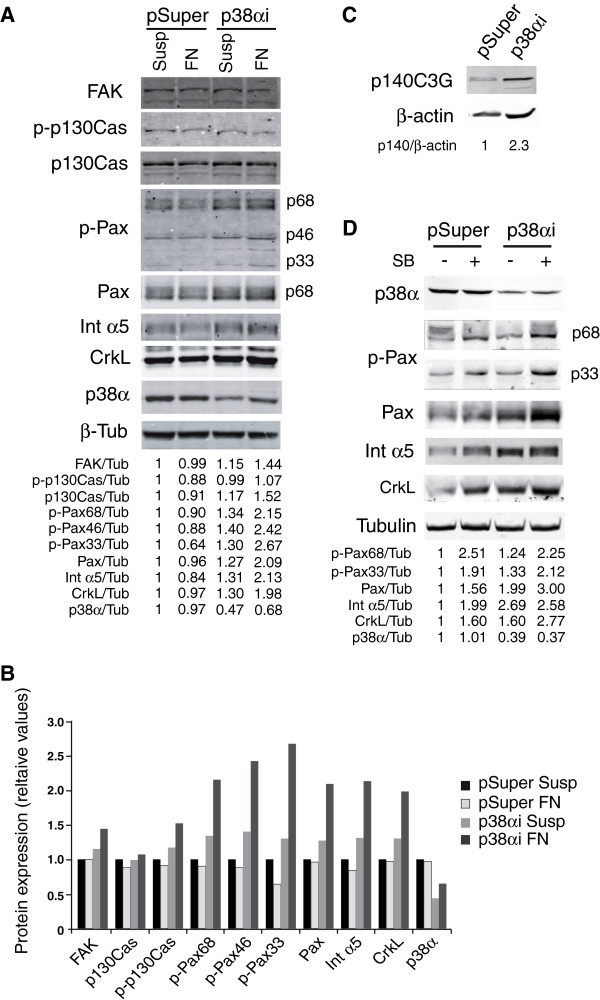
**p38α MAPK downregulate the expression and phosphorylation of FA proteins. (A)** Representative Western blots showing the expression and phosphorylation (p) of FAK, p130Cas, paxillin (Pax), CrkL, p38α and integrin α5 (Int α5) in K562 clones expressing pSuper-p38α MAPK (p38αi) or the empty vector (pSuper). Cells were cultured in suspension (Susp) or attached to fibronectin (FN). Relative ratios between the levels of the different analyzed proteins and ß-tubulin are shown. **(B)** The histogram represents the quantification by densitometry of the Western blot bands for each protein, relative to ß-tubulin. Susp: suspension; FN: fibronectin. (**C**) Western blot showing the expression of p140C3G in K562 cells expressing pSuper-p38α MAPK (p38αi) or empty vector (pSuper) grown on suspension. (**D**) Representative Western blots showing the expression and phosphorylation (p) of paxillin (Pax), CrkL, p38α and integrinα5 (Int α5) in K562 cells expressing pSuper-p38α MAPK (p38αi) or empty vector (pSuper) grown on suspension and treated or not with 5 μM SB203580 (SB) for 48 h. Relative ratios between the levels of the different analyzed proteins and ß-tubulin or ß-actin are shown.

Next, we wanted to analyze whether p38α MAPK plays a role in the regulation of the expression and activation of FA proteins. Figure 7A-B shows that p38α knock-down led to an upregulation of paxillin, integrin α5 and CrkL expression, especially in the presence of fibronectin, while FAK and p130Cas protein levels remained unchanged. C3G expression was also increased in p38α silenced cells (Figure 7C). In addition, p38α silencing induced an increase in the phosphorylation of all paxillin isoforms, which was more notorious in the presence of fibronectin. Similar results were obtained when cells were grown in the presence of SB203580 for 48 h, which even reinforced the effect of p38α silencing (7D). These results support an inhibitory effect of p38α on the expression and activation of FA proteins, in agreement with previous reports [18].

### Cbl, Abi1 and p130Cas regulate the expression and activation of FAK and paxillin in K562 cells

**Figure 8:**
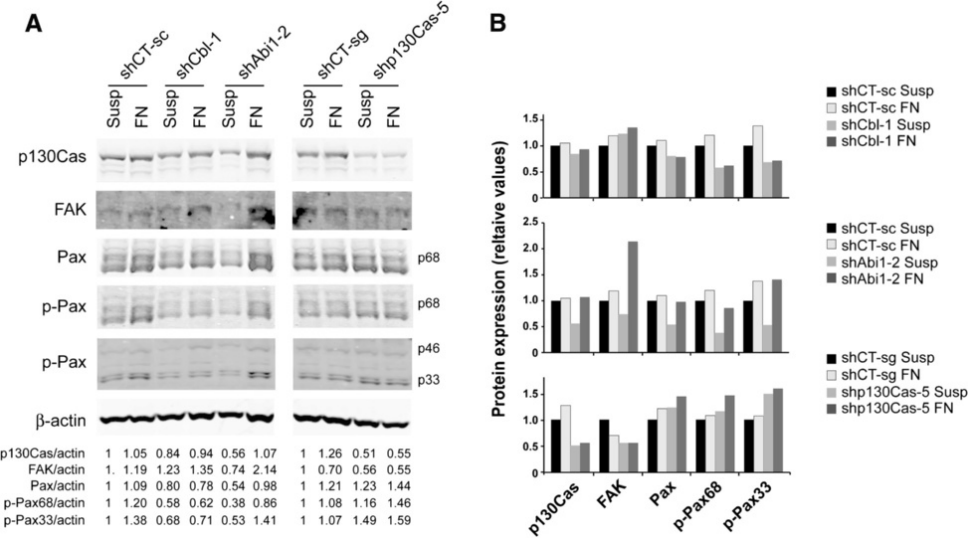
**Abi1, Cbl and p130Cas contribute to FA regulation. (A)** Representative Western blots showing the expression and phosphorylation (p) of the indicated proteins in K562 cells upon knock-down of Cbl (clone 1), Abi1 (clone 2) or p130Cas (clone 5), and their corresponding control cells (see Figure 3). ß-actin was used as loading control. Relative ratios between the levels of the different analyzed proteins and ß-actin are shown. **(B)** The histograms represent the relative values of the levels of expression of the analyzed proteins relative to ß-tubulin. shCT-sc: Control shRNA from Santa Cruz Biotech., shCT-sg: Control shRNA from Sigma. Susp: suspension; FN: fibronectin.

Finally, we have analyzed the contribution of adhesion proteins that interact with C3G and Bcr-Abl, such as Cbl, Abi1 and p130Cas, to the regulation of the expression and phosphorylation of FAK, p130Cas and paxillin, by knocking down these genes. The greatest effect was observed in Abi1 silenced cells maintained in suspension, where the protein levels of p130Cas, FAK and paxillin, as well as paxillin phosphorylation, decreased (Figure 8). These results are in agreement with the observed decrease in adhesion to fibronectin of Abi1 knock-down clones. This effect was reverted in the presence of fibronectin, where FAK expression was even increased, indicating that the expression of all these proteins is regulated by the fibronectin-triggered pathway. On the other hand, p130Cas knock-down led to an increase in the phosphorylation of paxillin, and to a slight decrease in FAK protein levels. In contrast, Cbl knock-down seems to have the opposite effect to that of p130Cas knock-down, as it reduces FAK levels and increases paxillin phosphorylation. In summary, these results are compatible with a negative role of p130Cas in CML cell adhesion and a positive role of Cbl, in agreement with the adhesion behavior to fibronectin of the respective silenced clones.

## Discussion

In this paper we have explored the nature of the interaction between C3G and Bcr-Abl proteins. This interaction requires Abi1 and p130Cas, while Cbl seems not to contribute, albeit it forms complexes with C3G. In this regard, a connection between c-Cbl and the regulation of cell migration and spreading through CrkL-C3G-Rap1 and Rac has been described [50]. Likewise, the interaction between Bcr-Abl and p130Cas seems to be more stable in the presence of Abi1 and Cbl. In contrast, CrkL competes with Abi1 in its binding to the SH3 domain of Abl.

The Abl-SH3 domain interacts with C3G and Abi1, as previously published [3,8,9]. In addition, we have uncovered a novel interaction between the Abl-SH3 domain and the Cbl-SH3-b domain. This has been demonstrated by i) pull-down assays and ii) far western analysis using arrays of purified SH3 domains, which indicate that this interaction is direct. A direct interaction between Cbl and the Abl-related protein Arg was also detected. Additionally, the Cbl-SH3-b domain can also establish an indirect interaction with Bcr-Abl, which involves Abl-Y177 and Grb2 [24]. Therefore, we describe a novel direct interaction, between Cbl and Bcr-Abl through their SH3-b and SH3 domains respectively, in addition to the direct Bcr-Abl-Cbl interaction, involving the Abl SH2 domain and Cbl phospho-tyrosines [24,51].

A direct association between p130Cas and the SH2 domains of Bcr-Abl or CrkL has been previously described [12]. Here we show that interactions involving the Bcr-Abl SH3 domain and the SH3 or SH3-b domains of p130Cas are also produced in CML cells. Moreover, the association between the p130Cas SH3-b and the Abl-SH3 domains could be direct, based on far Western experiments. Our results also illustrate a non-canonical association between p130Cas and p87C3G, which involves the proline-rich domain of p130Cas but not its SH3 domain. In this line, recent reports have assigned important roles to p130Cas SH3 and SH3-b domains in FA formation and sustained FA disassembling, respectively [52,53]. It is likely that the interaction between p87C3G and Bcr-Abl induces aberrant associations with FA molecules, thus, contributing to the adhesion defects observed in CML cells [15].

In addition, we have uncovered the existence of a series of new interactions, not described previously, between C3G, Bcr-Abl and FA proteins, such as Cbl, p130Cas, Abi1 and CrkL: (i) interaction between p87C3G and Abi1 SH3 or SH3-b domains; (ii) interaction of Cbl SH3-b domain with p87C3G; (iii) interaction between Bcr-Abl SH3 domain and CrkL. All these novel interactions strongly suggest the existence of complex networks between these proteins with dynamic connections involving multiple different domains. This reflects the complexity and plasticity of the regulation of the FA contacts in CML cells.

Different from our results derived from Two-Hybrid assays (unpublished data), SH3 arrays hybridization showed a weak interaction between C3G-SH3-b and Abl-SH3 domain (lower than the positive controls). This weak interaction detected *in vitro* may be not strong enough to be detected *in vivo*.

It is noteworthy that the SH3-b domains of C3G, Cbl and p130Cas show similar hybridization patterns with the SH3 domain arrays, which suggests that these three proteins are involved in the regulation of common signaling pathways. In fact, C3G, p130Cas and Cbl directly interact with proteins involved in FAs dynamics, in agreement with its participation in these complexes. Among them, we find Cortactin and Vinexin (VINE), protein tyrosine kinases such as Abl2 and c-Src, and adapter proteins such as CrkL. Immunoprecipitation assays confirmed previously described interactions between p-Paxillin and CrkL or p38α MAPK [44,45] and of CrkL with Bcr-Abl [46]. In addition, we also found a strong interaction between Abi1 and p130Cas, FAK and p38α MAPK and an interaction of p38α MAPK with C3G or CrkL. To our knowledge, these interactions have not been described so far.

In agreement with previous results [4,54], our data suggest that C3G plays a key role in the regulation of CML cell adhesion as (i) C3G silencing decreases adhesion to fibronectin, (ii) changes in C3G expression alters the levels of expression and activation of FA proteins, such as FAK, paxillin, CrkL, Cbl and integrin α5, and (iii) C3G silencing increases the interaction between CrkL and paxillin (difficult to observe in control cells) and decreases CrkL interaction with Bcr-Abl. This is relevant, since aberrant interaction between CrkL and paxillin is induced by Bcr-Abl in CML cells [46]. Therefore, p140C3G could act as a negative regulator of Bcr-Abl-induced abnormal adhesion. A role for C3G in the formation or stabilization of integrin β1- and paxillin-positive FAs has also been described in MEFs [54].

In agreement with other studies [55], Abi1 is a positive regulator of adhesion to fibronectin. In contrast, our results on Cbl are different from previous reports. Cbl negatively regulates cell adhesion in most systems by targeting α5-integrin, CrkL and FAK for ubiquitination [56,57]. However, our results reveal a positive role for Cbl in CML cell adhesion, as Cbl silencing impaired adhesion of CML cells. p130Cas seems to exert a negative effect in CML adhesion, in agreement with its role in migration and invasion [58,59]. Regarding p38α we have contradictory results as p38α knock-down decreased adhesion to fibronectin, but also increased the levels and/or activity of some FA proteins such as paxillin. The decreased adhesion observed in p38α silenced K562 cells point out to a positive regulation of CML adhesion by p38α, according to the role proposed for p38 in adhesion in human melanoma cells [60] and in Karpas 299 lymphoma cells based on the effect of the p38α/ß inhibitor SB203580 [61]. However, results derived from studies performed with p38α knock-out cells indicates that p38α plays a negative role in adhesion in mouse embryonic stem cells [18] and in embryonic cardiomyocytes [62]. Differences between cell types might account for these distinct functions of p38α in adhesion. It would be also possible that p38 could play a different role in normal and tumoral cells as adhesion is altered in tumoral cells.

In contrast to the reduced adhesion found in p38α silenced K562 cells, either p38α knockdown or SB203580 treatment induced an increase in the expression of FA proteins, mainly paxillin, integrin α5 and CrkL, as well as an increase in phospho-paxillin, which is normally associated with increased adhesion. This effect was stronger in cells attached to fibronectin. Similar results were observed by Guo and coworkers [18]. A plausible explanation is that the increase in the expression of FA proteins induced by p38α silencing alters the stoichiometry of the FA complexes, leading to adhesion defects likely due to the impairment of assembly and disassembly of focal adhesion complexes. Additionally, it should be noted that adhesion experiments were performed under serum-deprivation, which induces the activation of p38α and other p38 isoforms (mainly ß) [62,63] and can alter the activity of other signaling molecules involved in adhesion such as Rac1 [62]. All this would lead to a potential imbalance of different signaling pathways that could induce a decrease in adhesion. Finally, the fact that double C3G/p38α silenced cells present a similar decreased adhesion to fibronectin that the single knock-down clones, suggests that both proteins could participate in the same signaling pathway regulating cell adhesion. This is supported by the immunoprecipitation studies showing interaction between C3G and p38α MAPK. In addition, C3G and p38α MAPK interact with paxillin and FAK, indicating that they form complexes at the FA. Especially relevant is the interaction between p38α, paxillin and FAK, which strongly indicate that p38α stably interacts with these proteins.

The pathogenesis of CML is caused in part by disorders in the motility of CML cells as well as in their adherence to fibronectin and other substrates [15]. It has been suggested that Bcr-Abl interferes with the signaling normally induced by ß1 integrin activation, leading to a decrease in adhesion to fibronectin [14,64]. In fact, Bcr-Abl mimics integrin activation to establish aberrant interactions with paxillin [45]. There are evidences about the involvement of the SH3 domain of Bcr-Abl in the regulation of adhesion of leukemic cells through the formation of complexes with α2β1 integrin [13]. Moreover, interaction of Abi1/2 with the Bcr-Abl SH3 domain negatively regulates its kinase activity [31,32,65]. In fact, Abi1 triggers a downstream pathway, involving WAVE2 that contributes to Bcr-Abl-induced abnormalities in the cytoskeletal and integrin function [55]. Results presented herein suggest that Bcr-Abl function and consequently CML cell adhesion, could also be regulated by C3G, Cbl, p130Cas, CrkL and p38α MAPK through interactions involving the SH3 domain of Bcr-Abl. Supporting this idea, silencing of Abi1, C3G, Cbl, p130Cas and p38α regulate the expression and/or activation of FA proteins in CML cells. Moreover, p140C3G silencing decreases the Bcr-Abl/CrkL association and reinforces the abnormal interaction between CrkL and paxillin induced by Bcr-Abl, indicating that p140C3G negatively regulates the defective adhesion induced by Bcr-Abl.

## Conclusions

Our data indicate that C3G plays a relevant role in the regulation of adhesion in CML cells by interacting with Bcr-Abl, p38αMAPK, Cbl, p130Cas, Abi1, FAK and paxillin at the focal adhesions. It is likely that p140C3G levels in CML cells might be tightly controlled, as either its overexpression or downregulation induce a decrease in the protein levels of key FA proteins, such as paxillin and FAK. A similar behavior was observed in the regulation of apoptosis in CML cells [16]. In this line, it is plausible to hypothesize that the C3G isoform, p87C3G, participates in the perturbation of the adhesive properties of the CML cells through interaction with the Bcr-Abl-SH3 domain (regulator of the adhesion) and the establishment of aberrant associations with FA proteins, thus avoiding their normal interaction with other proteins, including p140C3G. Further investigation is warranted to ascertain the relationship between p140C3G and p87C3G in the regulation of CML adhesion.

Our data also support a role for p38α in cell adhesion in CML as p38α knock-down decreases adhesion to fibronectin and changes the levels and/or phosphorylation state of some FA proteins. In addition, because silencing of either p38α and/or C3G induced a similar reduction in adhesion, p38α might be acting in the C3G pathway. Future studies will uncover the precise function of p140C3G, p87C3G and p38α in the regulation of all the different proteins involved in adhesion.

## Materials and methods

### Cell lines and expression constructs

K562 (ATCC, CCL243), a human cell line derived from a patient with CML in terminal blast crisis, was maintained in RPMI 1640 containing 10% fetal bovine serum (FBS). C3G overexpressing construct, pLTR2C3G, and constructs containing shRNAs to target either human C3G or p38α genes using pSuper.neo+gfp vector (Oligoengine) have been described previously [16,66].

### K562 infection with shRNA lentiviral particles

Expression of C3G, Abi1 and Cbl was silenced in K562 cells by transfection of (h) Lentiviral Particles: C3G shRNA (sc-29863-V), Abi1 shRNA (sc-40306-V), Cbl shRNA (sc-29242-V) and control shRNA (sc-108080) from Santa Cruz Biotechnology, following the manufacturer`s protocol. For p130Cas silencing we used p130Cas shRNA (MISSION® Transduction particles NM_014567) and control shRNA (MISSION® pLKO.1-puro Empty Vector Control Transduction Particles) from Sigma. Puromycin-resistant clones were selected after 15 days in culture in 10%FBS/RPMI media supplemented with 2 μg/ml puromycin.

### Antibodies

Antibodies against: C3G (C-19, G4 and H-300), CrkL (C-20), Bcr (G6), c-Abl (2411)–, Cbl (C-15), Abi1 (H-80), p130Cas (35B.1A4), Grb2 (C-23), GST (B-14), p38α (C-20) and integrin α5 (H-104), p-FAK (ser 722) were from Santa Cruz Biotechnology; β-Tubulin clone 2-28-33 and Actin, clone MM2/193 were from Sigma-Aldrich; FAK, phospho-p130Cas (Tyr249) and phospho-CrkL (Tyr207) were from Cell Signaling Technology; paxillin (clone 349) was from BD Biosciences; phospho-paxillin (Y118) was purchased from Life Technologies. Anti-His_6_-Peroxidase was from Roche. Anti-Abi1 antibody (SSH3BP1) is a monoclonal antibody from Abcam (#ab11222). C3G-1008 serum [66] was used in the immunofluorescence experiments.

As secondary antibodies we used: Cy3 anti-rabbit, Cy3 anti-mouse, Cy5 anti-rabbit, Alexa Fluor 488 anti-goat, FITC anti-rabbit, and FITC anti-mouse from Jackson ImmunoResearch Laboratories, Inc. For F-actin detection we used Oregon Green® 514 phalloidin from Life Technologies.

### Cell adhesion assay

Adhesion of K562 cells infected with shRNA lentivirus (knock-down) was performed as described [67]. Briefly, 96-well plates were coated with fibronectin at 50 μg/ml in PBS and then blocked by the addition of BSA 1%. Cells were washed with PBS and resuspended in medium without serum at 5×10^6^cells/ml. Then, cells were labeled with Calcein AM fluorescence dye (BD Biosciences) according to manufacturer`s instructions and added to each microplate well in 100 μl at 5×10^6^ cells/ml. After 3 h at 37°C, plates were washed 3 times with medium and inverted onto filter paper to blot excess liquid. The remaining calcein-labeled cells were suspended in PBS and used to determine the total of cells added. Adhesion was measured in a fluorescence plate reader (ULTRA Evolution; Serial number: 12903200010; Firmware: E 1.03 02/03 EVOLUTION; XFLUOR4 Version: V 4.50). The percentage of adhesion was determined by dividing the corrected (background subtracted) fluorescence of adherent cells by the total corrected fluorescence of cells added to each microplate well.

### Immunoblotting

Whole cell lysates were prepared using cell lysis buffer (20 mM Tris–HCl pH 7.5, 150 mM NaCl, 1% Triton X-100 (or NP-40), 0.1% Na deoxycholate, 0.1% SDS) supplemented with 1 mM PMSF, 10 μg/ml Aprotinin and 10 μg/ml Leupeptin. Cell debris was removed by spinning at 10000 *g* for 10 min at 4°C.

### Immunoprecipitation

Immunoprecipitation was performed as described [68]. Prior to the immunoprecipitation, lysates were precleared by incubation with washed GammaBind G Sepharose beads (GE Healthcare Life Sciences) for 30 minutes.

### Pull-down assays

Constructs: Abi1-SH3, Abi1-SH3-b (SH3-binding), Cbl-SH3-b and p130Cas-SH3, p130Cas-P1 (proline-rich domain 1 or SH3-b1) and p130Cas-P2 (SH3-b2) domains were expressed as GST-fusion proteins. To do so, fragments were amplified by PCR and cloned into *Eco*RI-*Xho*I sites of pGEX-4T-1 (GE Healthcare Life Sciences). The oligos used were Abi1SH3-F: 5´-GGGGAATTCCCCAAGAATTATATTGAGAAAGTT-3´ and Abi1SH3-R: 5´-GGGCTCGAGTTAATCAGTATAGTGCATGATTGA-3; Abi1SH3b-F: 5´-GGGGAATTCCCCATTGCTGTGCCTACA-3´ and Abi1SH3b-R: 5´-GGGCTCGAGCAGCCTCCTCATCTTCAT-3´; CblSH3b-F: 5´-GAGGAATTCCCGCCTTCTCCATTCTCC-3´ and CblSH3b-R: 5´-GGGCTCGAGAGGTGGCAGTTTTGGCAC-3´; p130CasSH3-F: 5´-AGGGAATTCAACCACCTGAACGTGCTG-3´ and p130CasSH3-R: 5´-AGGCTCGAGGCCCACCAAGATCTTGAG-3; p130CasP1-F: 5´-AGGGAATTCGATAAGAAGCCAGCAGGG-3 and p130CasP1-R: 5´-AGGCTCGAGGTAGACGCTGTCTGGCTG-3´; p130CasP2-F: 5´-AGGGAATTCTCACTGCTCTTCAGACGG-3´ and p130CasP2-R: 5´-GGGCTCGAGGGTGAACTTAGGGGGTGA-3´. GST-Abl-SH3 and GST-C3G-SH3-b constructs have been described previously [8].

Pull-downs were carried out by incubating 1 mg of protein extract in lysis buffer with 12 –μg of GST-fusion proteins, bound to glutathione-sepharose 4 fast flow beads (GE Healthcare Life Sciences), for 2 hours at 4°C. Complexes were subjected to 3 washes in lysis buffer and boiled in loading buffer before SDS- PAGE.

### Immunofluorescence

Immunofluorescence was performed essentially as described [68].

### SH3 domain arrays

Panomics´ SH3 domain Arrays are designed to determine whether a protein of interest binds to multiple SH3 domains (Panomics). Recombinant proteins of interest (C3G-SH3-b, Cbl-SH3-b or p130Cas-SH3-b domains) were engineered with a N-Terminal His-Tag by cloning the corresponding fragments into the pET15b-derived plasmids (Novagen-EMD Millipore) pETEV15b [69] and pET15b-NBKSXa (Additional file 11: Methods 2 and Additional file 12: Method 3). Proteins were expressed in *E. coli* strain BL21 (DE3) and purified by affinity chromatography as described [70]. Purified proteins were hybridized with the SH3 Domain Array I (Panomics, Cat #MA3010), and Array II (Panomics, Cat #MA3011), following the manufacturer`s protocol. Hybridizations were visualized with peroxidase-conjugated anti-His antibodies, followed by ECL plus (Amersham). Spots with intensities similar or stronger than positive controls (pos) were selected as ligands of the corresponding SH3-b domain.

### Image processing

Quantification of band intensity was performed by Image J version 1.24 software [71].

### Statistical analysis

Data are represented as mean ± SEM. Statistical analysis was performed using an unpaired Student's t-test. Results were considered significant when p<0.05(*).

## Competing interests

The authors declare that they have no competing interests.

## Authors’ contributions

VM carried out the pull-down experiments, VM and SO-R made the immunofluorescences, immunoprecipitations and cell adhesion assays, MS and JG-B made all the constructs, VM and MS performed the shRNA lentiviral constructs, VM and IF-A participated in the SH3 arrays hybridizations, VM and SG-H performed the immunoblottings, CG, JMP and AP designed the experiments, CG wrote the paper, AP and JMP critically revised the paper. All authors read and approved the final manuscript.

## Supplementary Material

Additional file 1 **Comparative expression of C3G isoforms p140C3G and p87C3G in whole cell lysates from NIH 3T3, Boff210 and K562 cells.** Boff210 are BaF/3-derived cells expressing the Bcr-Abl oncogene [8]. Click here for file

Additional file 2 **C3G forms immunocomplexes with Bcr-Abl, p130Cas, Cbl and Abi1.** Immunoprecipitates of K562 lysates with the indicated antibodies. Immunoblotted proteins are indicated on the margin. Antibodies used in the immunoprecipitations and immunoblotting are described in the manuscript. IP: immunoprecipitation; IB: immunoblotting; B: γ-bind sepharose beads; L: whole cell lysate. Click here for file

Additional file 3 **Abl**-**SH3 domain interacts with CrkL by an indirect mechanism.(A)** Detection of CrkL by pull-down assay in K562 lysates, using the Abl-SH3 domain fused to GST as bait. **(B)** CrkII and CrkL SH2-domain sequence alignment. The internal SH3-b domain within the CrkII SH2 domain is overlined with a double arrow. The putative proline-rich motifs are shadowed. **(C)** Pull-down assays in K562 lysates using the CrkL SH2 or SH3-N domains fused to GST as baits. Expression of Bcr-Abl, p140C3G and p87C3G was detected by immunoblotting with antibodies against Bcr and C3G (C-19) respectively. **(D)** Two-Hybrid analysis of whole CrkL, CrkL-SH2 or CrkL-SH3-N domains, cloned into pSos, and Abl-SH3 domain cloned in pMyr. PD: pull-down. L: whole cell lysate. Click here for file

Additional file 4: Method 1 Two-Hybrid. To detect direct interactions between CrkL-SH2 and Bcr-Abl-SH3 domains we used the Two-Hybrid System CytoTrap® Vector Kit (Agilent Technologies, formerly Stratagene) following the manufacturer`s indications. CrkL-SH2 domain was amplified with oligos CrkL-F: 5’-TTTGGATCCATGTCCTCCGCCAGGTT-3 and CrkL SH2-R: 5’-TTTGAATTCTCATGGGTGCTGAGACAGATC-3’; CrkL-SH3-N domain was amplified with oligos CrkL-SH3-F: 5’-AAAGGA TCCGATCTGTCTCAGCACCCA-3’ and CrkL-SH3-R: 5’-TTTGAATTCTCAAGCAGGTTCTGGGATCC-3’. Whole CrkL c-DNA was amplified with oligos CrkL-F (see above) and CrkL-R: 5’-TTTGAATTCTCACTCGTTTTCATCTGGGT-3’. All CrkL fragments were cloned into pSos plasmid. Abl-SH3 domain was amplified with oligos Abl-SH3-F: 5´-CCCGAATTCTTTCTGAATGTCATCGTCC-3´ and Abl-SH3-R: 5´-CCCCTCGAGAGAAGCTGCCATTGATCC-3´ and cloned into pMyr plasmid. Click here for file

Additional file 5 **Western blots of arrays I and II hybridized with His-tagged-C3G-SH3-b domain and developed with anti-His antibodies.** Each pair of dots represent an immobilized SH3 domain fused to GST (see details in Materials and Methods). Dots labeled as (pos) are histidine ligands which are used as positive controls for the hybridization and detection. GST dots are the negative controls. Click here for file

Additional file 6 **Western blots of arrays I and II hybridized with His-tagged-Cbl-SH3-b domain and developed with anti-His antibodies.** Each pair of dots represent an immobilized SH3 domain fused to GST (see details in Materials and Methods). Dots labeled as (pos) are histidine ligands which are used as positive controls for the hybridization and detection. GST dots are the negative controls. Click here for file

Additional file 7 **Western blots of arrays I and II hybridized with His-tagged-p130Cas-P2 domain and developed with anti-His antibodies.** Each pair of dots represent an immobilized SH3 domain fused to GST (see details in Materials and Methods). Dots labeled as (pos) are histidine ligands which are used as positive controls for the hybridization and detection. GST dots are the negative controls. Click here for file

Additional file 8: Table S1 SH3 domain list Array I. Click here for file

Additional file 9: Table S2 SH3 domain list Array II. Click here for file

Additional file 10 **Histogram representing the percentage of cell adhesion to laminin, fibronectin or collagen of K562 clones stably transfected with lentiviral particles to silence C3G expression (shC3G-1) or with shRNA control (shCT).** *p<0.05 versus shCT. Click here for file

Additional file 11: Method 2 pET15b-NBKSXa vector cloning region. pET15b-NBKSXa cloning/expression region (modified from pET15b) indicating the histidine tag and the thrombin recognition sequence and cleavage site. Click here for file

Additional file 12: Method 3 Cloning of C3G-SH3-b, Cbl-SH3-b or p130Cas-SH3-b domains into pETEV15b or pET15b-NBKSXa vectors. C3G-SH3-b domain was amplified by PCR with oligos C3GSH3-bF: 5´-GGGGAATTCCCATGGCTGGCATTCGGGTGGTTGAT-3´ and C3GSH3-bR: 5´-CCCGGATCCCTACTAACTGCCGTCTCTGCTGTCC-3´ and cloned into *Nco*I-*Bam*HI sites of pETEV15b. Cbl-SH3-b domain was amplified with oligos CblSH3bBamHI-F:5´-GGGGGATCCCCGCCTTCTCCATTCTC-3´ and CblSH3bXhoI-R: 5´-CCCCTCGAGCTACTAAGGTGGCAGTTTTGGCAC-3´ and cloned into pET15b-NBKSXa by *Bam*HI-*Xho*I digestion. p130CasP2-domain (proline-rich region 2) was amplified with oligos CasP2BamHI-F: 5´-AGGGGATCCTCACTGCTCTTCAGACGG-3 and CasP2XhoI-R: 5´-GGGCTCGAGCTACTAGGTGAACTTAGGGGGTGA-3´ and cloned into *Bam*HI-*Xho*I sites of pET15b-NBKSXa. Click here for file
