# Supplementary figures and images for: C3G forms complexes with Bcr-Abl and p38α MAPK at the focal adhesions in chronic myeloid leukemia cells: implication in the regulation of leukemic cell adhesion
Source: Cell Commun Signal. 2013 Jan 23;11:9. doi: 10.1186/1478-811X-11-9 (PMC3629710; doi:10.1186/1478-811X-11-9)

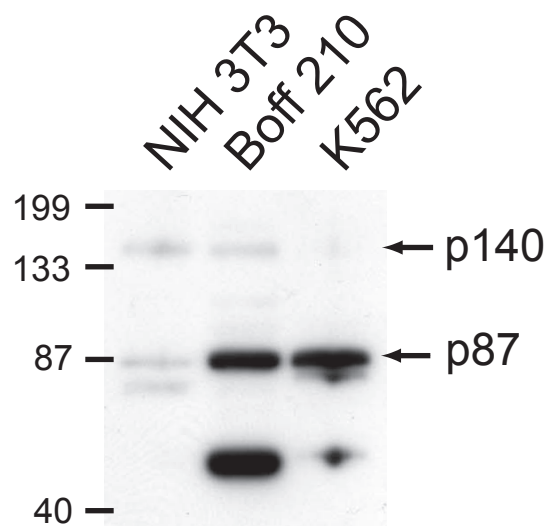

IB: C3G (c-19)

Supplement: Additional file 1 — Comparative expression of C3G isoforms p140C3G and p87C3G in whole cell lysates from NIH 3T3, Boff210 and K562 cells. Boff210 are BaF/3-derived cells expressing the Bcr-Abl oncogene [8]. [file 1478-811X-11-9-S1.pdf]

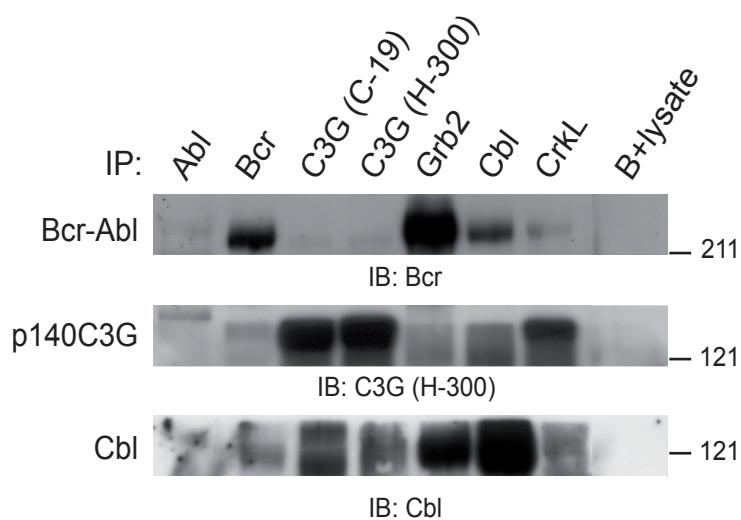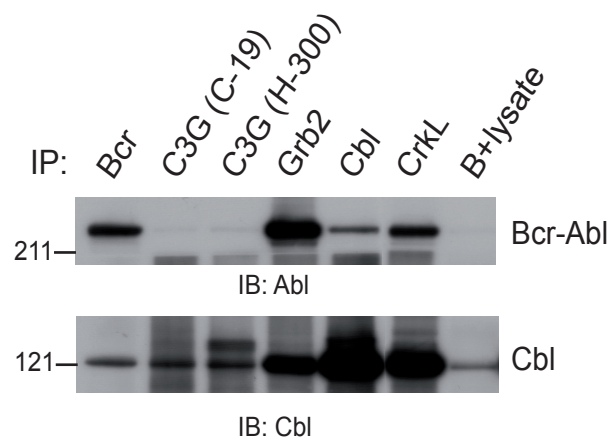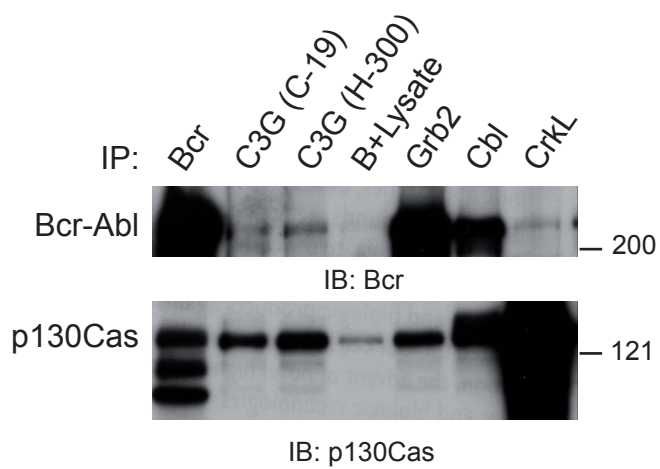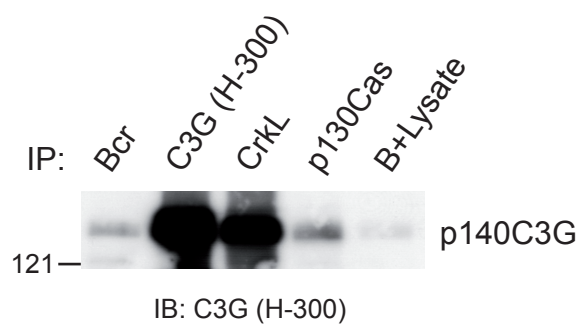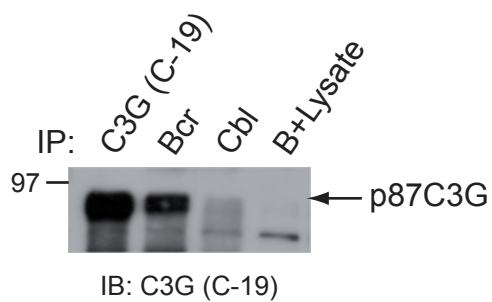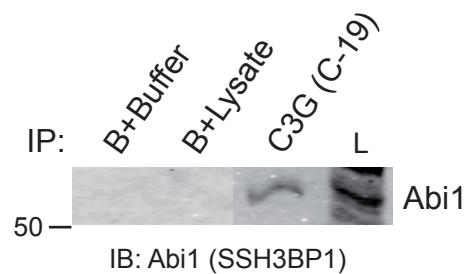

Supplement: Additional file 2 — C3G forms immunocomplexes with Bcr-Abl, p130Cas, Cbl and Abi1. Immunoprecipitates of K562 lysates with the indicated antibodies. Immunoblotted proteins are indicated on the margin. Antibodies used in the immunoprecipitations and immunoblotting are described in the manuscript. IP: immunoprecipitation; IB: immunoblotting; B: γ-bind sepharose beads; L: whole cell lysate. [file 1478-811X-11-9-S2.pdf]

C3G-SH3-b domain

Array I

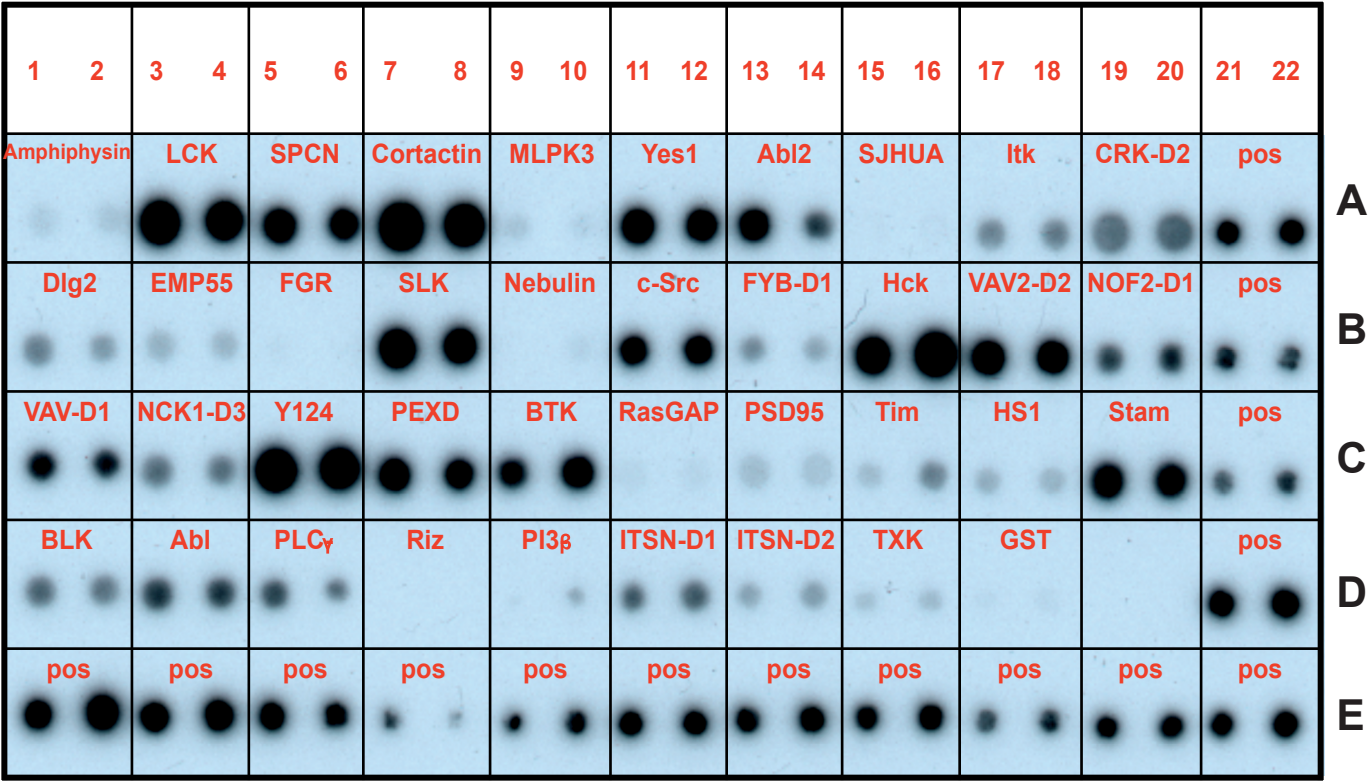

Array II

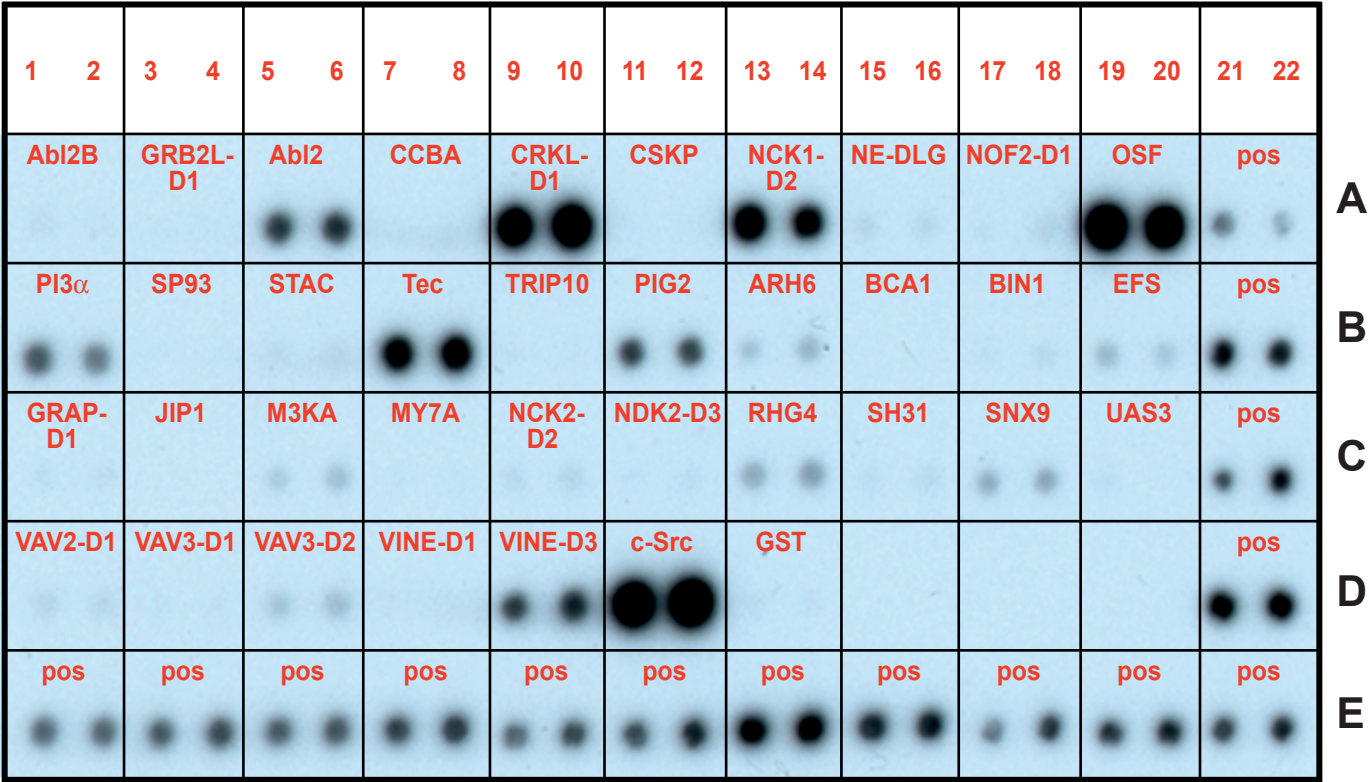

Supplement: Additional file 5 — Western blots of arrays I and II hybridized with His-tagged-C3G-SH3-b domain and developed with anti-His antibodies. Each pair of dots represent an immobilized SH3 domain fused to GST (see details in Materials and Methods). Dots labeled as (pos) are histidine ligands which are used as positive controls for the hybridization and detection. GST dots are the negative controls. [file 1478-811X-11-9-S5.pdf]

Cbl-SH3-b domain

Array I

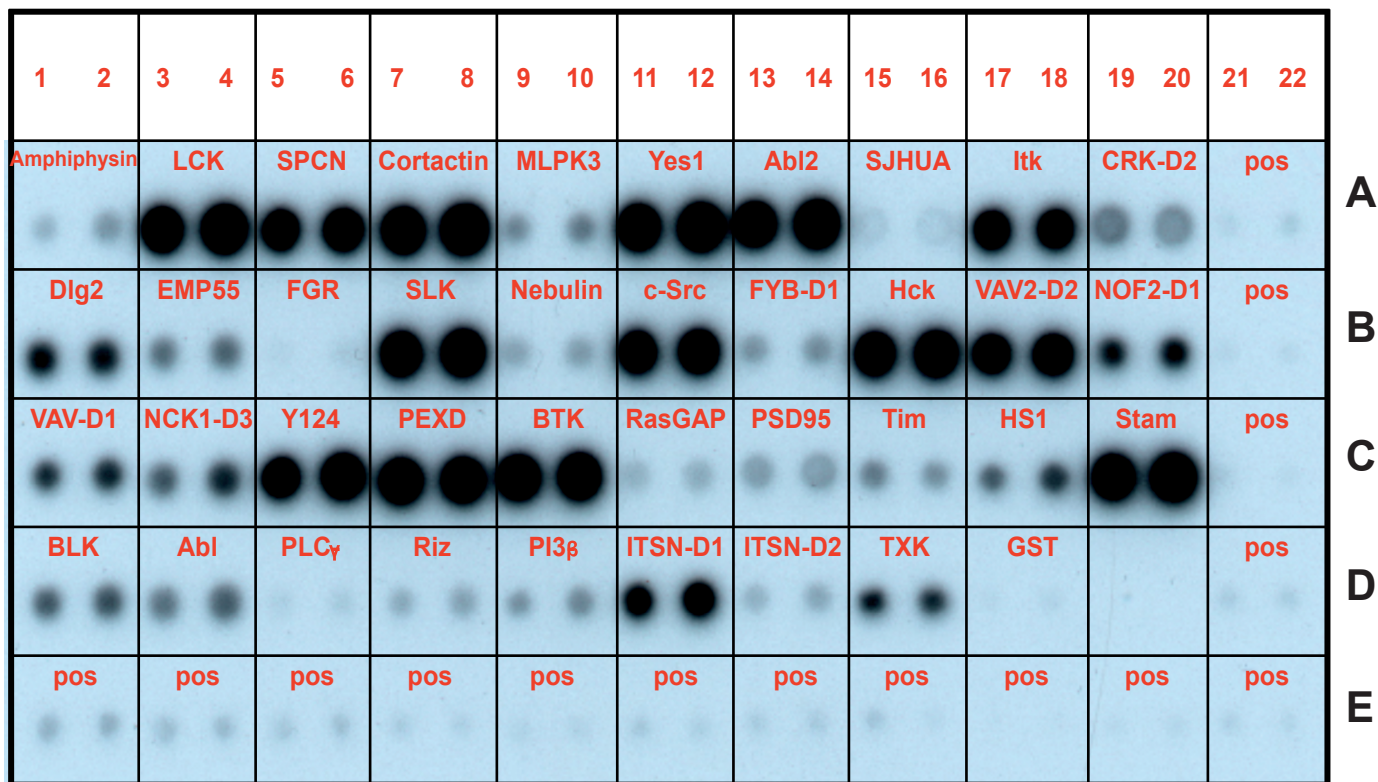

Array II

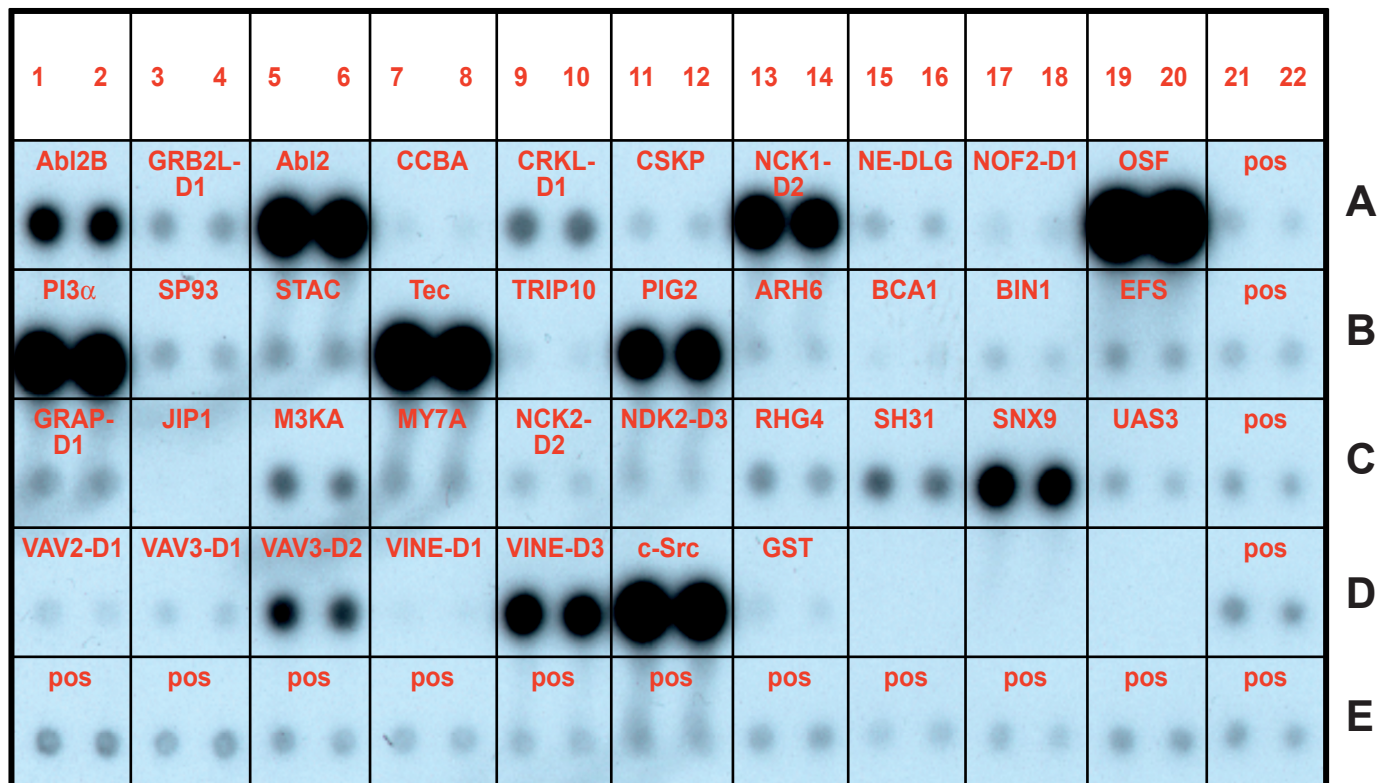

Supplement: Additional file 6 — Western blots of arrays I and II hybridized with His-tagged-Cbl-SH3-b domain and developed with anti-His antibodies. Each pair of dots represent an immobilized SH3 domain fused to GST (see details in Materials and Methods). Dots labeled as (pos) are histidine ligands which are used as positive controls for the hybridization and detection. GST dots are the negative controls. [file 1478-811X-11-9-S6.pdf]

**p130Cas-SH3-b (P2) domain**

**Array I**

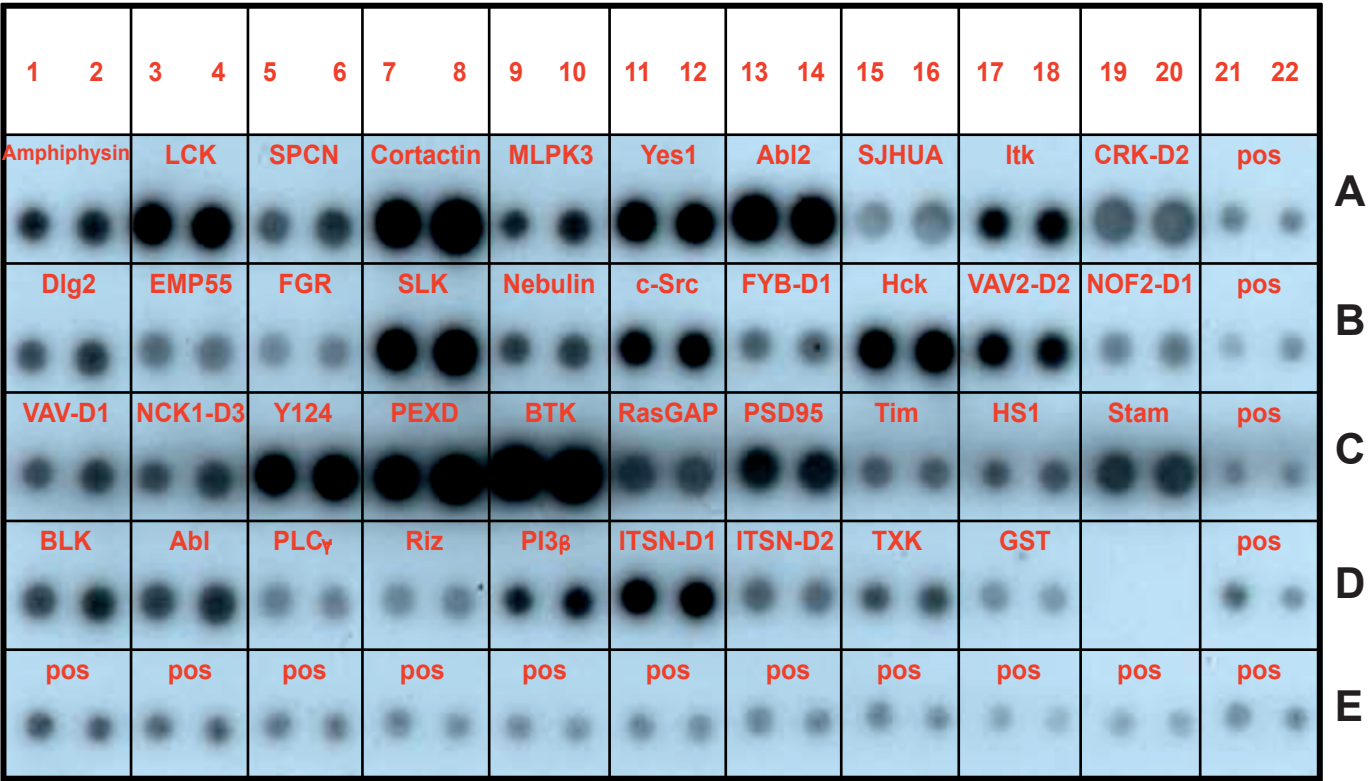

**Array II**

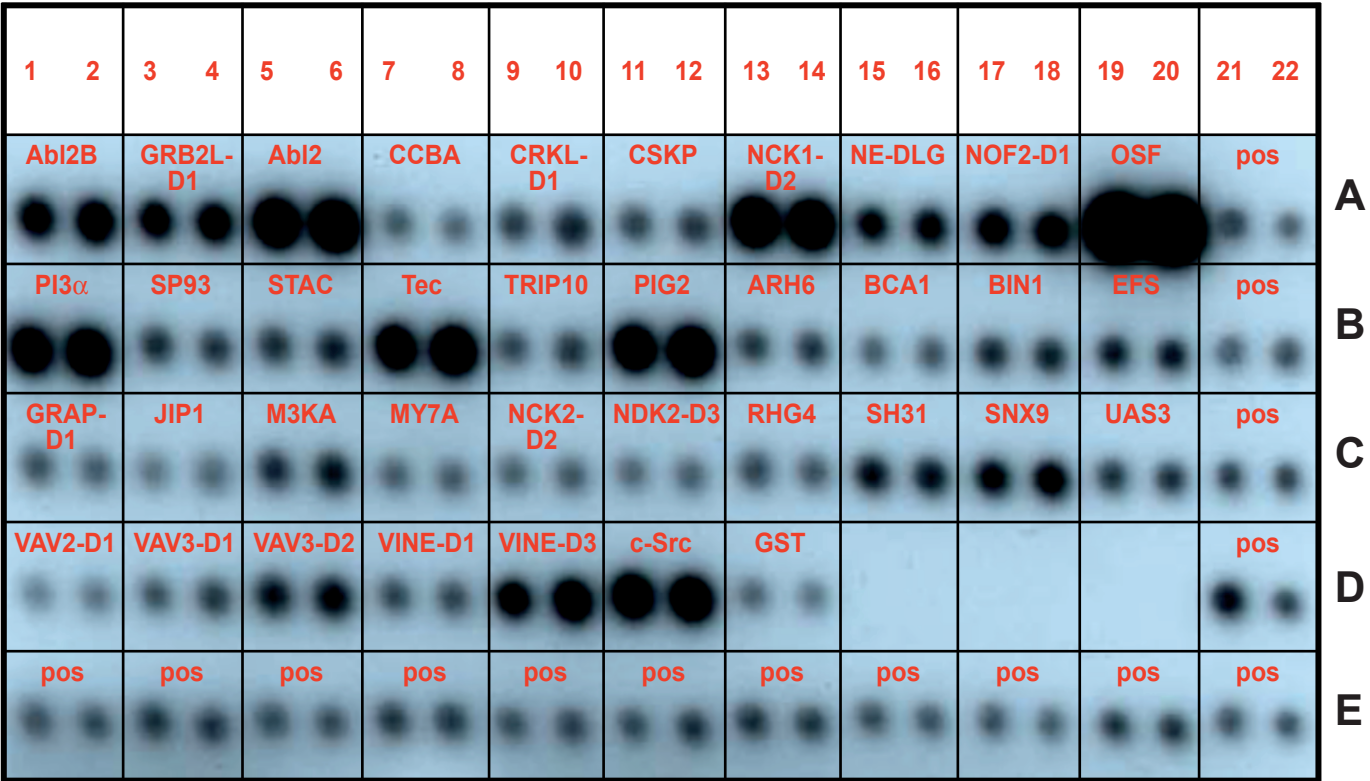

Supplement: Additional file 7 — Western blots of arrays I and II hybridized with His-tagged-p130Cas-P2 domain and developed with anti-His antibodies. Each pair of dots represent an immobilized SH3 domain fused to GST (see details in Materials and Methods). Dots labeled as (pos) are histidine ligands which are used as positive controls for the hybridization and detection. GST dots are the negative controls. [file 1478-811X-11-9-S7.pdf]

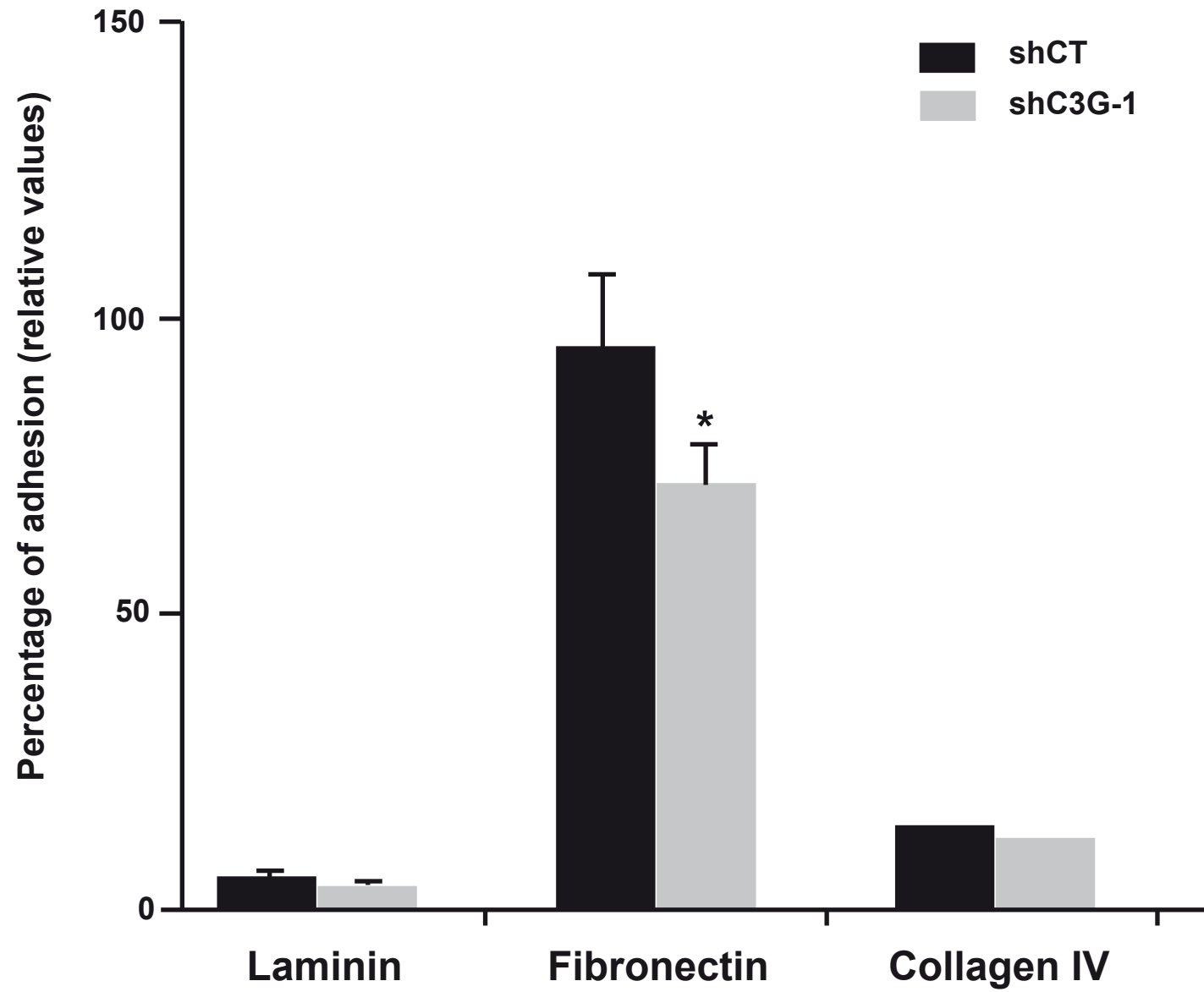

Supplement: Additional file 10 — Histogram representing the percentage of cell adhesion to laminin, fibronectin or collagen of K562 clones stably transfected with lentiviral particles to silence C3G expression (shC3G-1) or with shRNA control (shCT). *p<0.05 versus shCT. [file 1478-811X-11-9-S10.pdf]
