# Supplementary material for: C3G forms complexes with Bcr-Abl and p38α MAPK at the focal adhesions in chronic myeloid leukemia cells: implication in the regulation of leukemic cell adhesion
Source: Cell Commun Signal. 2013 Jan 23;11:9. doi: 10.1186/1478-811X-11-9 (PMC3629710; doi:10.1186/1478-811X-11-9)
Supplement: Additional file 3 — Abl-SH3 domain interacts with CrkL by an indirect mechanism.(A) Detection of CrkL by pull-down assay in K562 lysates, using the Abl-SH3 domain fused to GST as bait. (B) CrkII and CrkL SH2-domain sequence alignment. The internal SH3-b domain within the CrkII SH2 domain is overlined with a double arrow. The putative proline-rich motifs are shadowed. (C) Pull-down assays in K562 lysates using the CrkL SH2 or SH3-N domains fused to GST as baits. Expression of Bcr-Abl, p140C3G and p87C3G was detected by immunoblotting with antibodies against Bcr and C3G (C-19) respectively. (D) Two-Hybrid analysis of whole CrkL, CrkL-SH2 or CrkL-SH3-N domains, cloned into pSos, and Abl-SH3 domain cloned in pMyr. PD: pull-down. L: whole cell lysate. [file 1478-811X-11-9-S3.pdf]

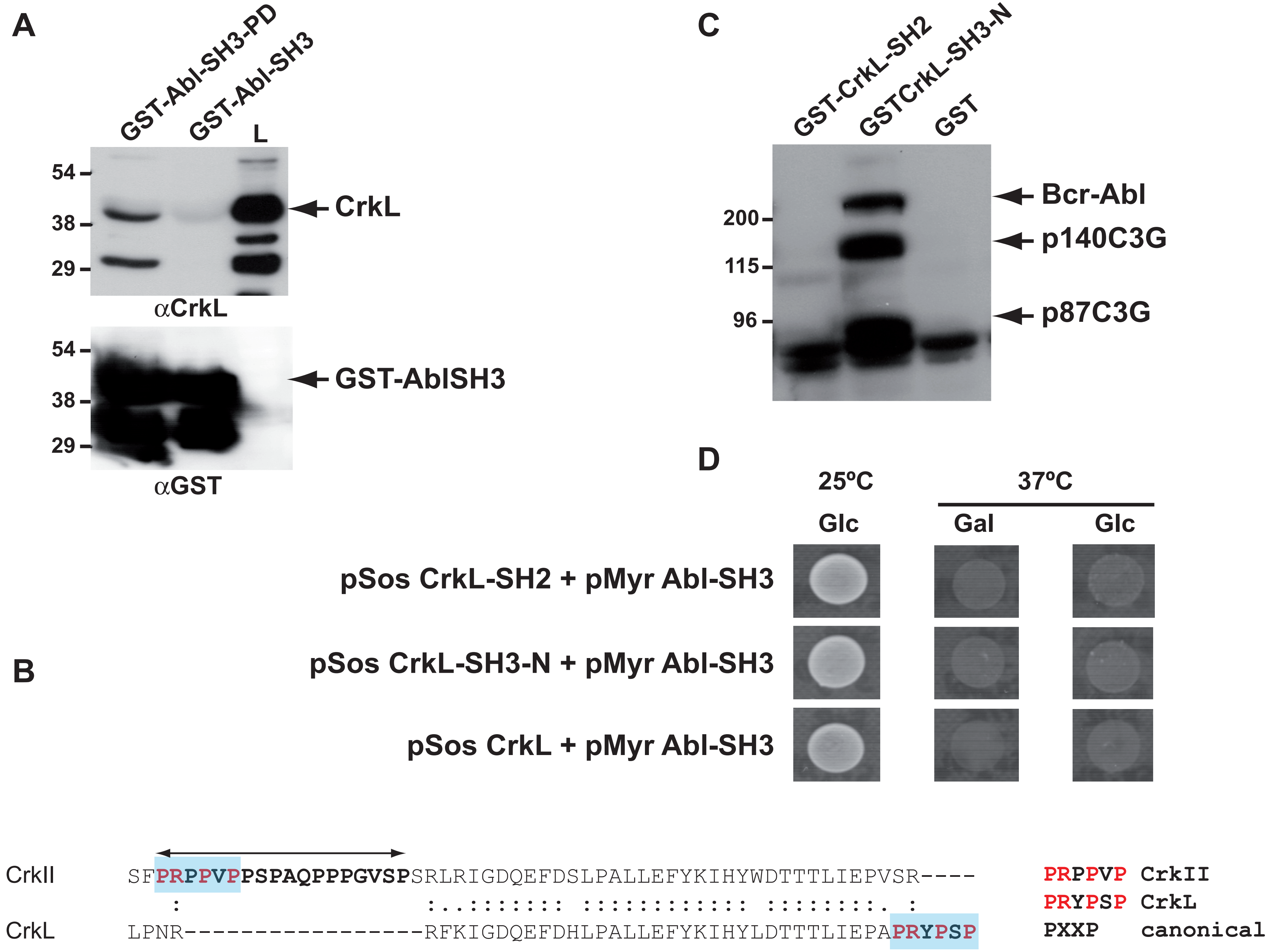

Additional file 3
