# Supplementary material for: C3G forms complexes with Bcr-Abl and p38α MAPK at the focal adhesions in chronic myeloid leukemia cells: implication in the regulation of leukemic cell adhesion
Source: Cell Commun Signal. 2013 Jan 23;11:9. doi: 10.1186/1478-811X-11-9 (PMC3629710; doi:10.1186/1478-811X-11-9)
Supplement: Additional file 4: Method 1 — Two-Hybrid. To detect direct interactions between CrkL-SH2 and Bcr-Abl-SH3 domains we used the Two-Hybrid System CytoTrap® Vector Kit (Agilent Technologies, formerly Stratagene) following the manufacturer`s indications. CrkL-SH2 domain was amplified with oligos CrkL-F: 5’-TTTGGATCCATGTCCTCCGCCAGGTT-3 and CrkL SH2-R: 5’-TTTGAATTCTCATGGGTGCTGAGACAGATC-3’; CrkL-SH3-N domain was amplified with oligos CrkL-SH3-F: 5’-AAAGGA TCCGATCTGTCTCAGCACCCA-3’ and CrkL-SH3-R: 5’-TTTGAATTCTCAAGCAGGTTCTGGGATCC-3’. Whole CrkL c-DNA was amplified with oligos CrkL-F (see above) and CrkL-R: 5’-TTTGAATTCTCACTCGTTTTCATCTGGGT-3’. All CrkL fragments were cloned into pSos plasmid. Abl-SH3 domain was amplified with oligos Abl-SH3-F: 5´-CCCGAATTCTTTCTGAATGTCATCGTCC-3´ and Abl-SH3-R: 5´-CCCCTCGAGAGAAGCTGCCATTGATCC-3´ and cloned into pMyr plasmid. [file 1478-811X-11-9-S4.doc]

**Additional method 1. Two-Hybrid**

To detect direct interactions between CrkL-SH2 and Bcr-Abl-SH3 domains we used the Two-Hybrid System CytoTrap® Vector Kit (Agilent Technologies, formerly Stratagene) following the manufacturer`s indications. CrkL-SH2 domain was amplified with oligos CrkL-F: 5’-TTTGGATCCATGTCCTCCGCCAGGTT-3 and CrkL SH2-R: 5’-TTTGAATTCTCATGGGTGCTGAGACAGATC-3’; CrkL-SH3-N domain was amplified with oligos CrkL-SH3-F: 5’-AAAGGA TCCGATCTGTCTCAGCACCCA-3’ and CrkL-SH3-R: 5’-TTTGAATTCTCAAGCAGGTTCTGGGATCC-3’. Whole CrkL c-DNA was amplified with oligos CrkL-F (see above) and CrkL-R: 5’-TTTGAATTCTCACTCGTTTTCATCTGGGT-3’. All CrkL fragments were cloned into pSos plasmid. Abl-SH3 domain was amplified with oligos Abl-SH3-F: 5´-CCCGAATTCTTTCTGAATGTCATCGTCC-3´ and Abl-SH3-R: 5´-CCCCTCGAGAGAAGCTGCCATTGATCC-3´ and cloned into pMyr plasmid.
