# Supplementary material for: C3G forms complexes with Bcr-Abl and p38α MAPK at the focal adhesions in chronic myeloid leukemia cells: implication in the regulation of leukemic cell adhesion
Source: Cell Commun Signal. 2013 Jan 23;11:9. doi: 10.1186/1478-811X-11-9 (PMC3629710; doi:10.1186/1478-811X-11-9)
Supplement: Additional file 8: Table S1 — SH3 domain list Array I. [file 1478-811X-11-9-S8.doc]

| Additional Table 1. SH3 domain list Array I | | |
| --- | --- | --- |
| Position | Domain | Full name |
| A1, 2  B1, 2  C1, 2  D1, 2  A3, 4  B3, 4  C3, 4  D3, 4  A5, 6  B5, 6  C5, 6  D5, 6  A7, 8  B7, 8  C7, 8  D7, 8  A9, 10  B9, 10  C9, 10  D9, 10  A11, 12  B11, 12  C11, 12  D11, 12  A13, 14  B13, 14  C13, 14  D13, 14  A15, 16  B15, 16  C15, 16  D15, 16  A17, 18  B17, 18  C17, 18  D17, 18  A19, 20  B19, 20  C19, 20 | Amphiphysin  Dlg2  VAV-D1  BLK  LCK  EMP55  NCK1-D3  Abl  SPCN  FGR  Y124  PLCg  Cortactin  SLK  PEXD  Riz  MLPK3  Nebulin  BTK  PI3b  Yes1  c-Src  RasGAP  ITSN-D1  Abl2  FYB-D1  PSD95  ITSN-D2  SJHUA  Hck  Tim  TXK  Itk  VAV2-D2  HS1  control  CRK-D2  NOF2-D1  Stam | Amphiphysin  Discs large homolog 2  VAV proto-oncogene, SH3 domain #1  Beta-lymphocyte specific protein tyrosine kinase  Human T-lymphocyte specific protein tyrosine kinasep56 Lck  55 kDa erythrocyte membrane protein  Cytoplasmic protein NCK1, SH3 domain #3  Abelson tyrosine kinase  Spectrin alpha chain (non-erythrocytic)  Cellular Gardner-Rasheed feline sarcoma virusprotein  PAK-interacting exchange factor beta  Phospholipase C gamma-1  Cortactin  Proto-oncogene tyrosine protein kinase  Peroxisomal membrane protein PEX13  Retinoblastoma protein-interacting zinc-finger  Mixed-lineage kinase 3  Nebulin  Bruton Tyrosine Kinase  Phosphoinositide-3-kinase regulatory beta subunit  Yamaguchi sarcoma virus oncogene homolog 1  Cellular Rous Sarcoma viral oncogene homolog  Ras GTPase-activating protein 1  Intersectin, SH3 Domain #1  Abelson-related protein; Arg  Fyn binding protein, SH3 domain #1  Presynaptic density protein 95  Intersectin 1, SH3 Domain #2  Spectrin alpha chain, erythrocyte  Hemopoietic cell kinase  Rho guanine nucleotide exchange factor (GEF) 5  Tyrosine-protein kinase TXK  Interleukin-2-inducible T-cell kinase  Vav2 oncogene product, SH3 Domain #2  Hematopoietic specific protein 1  Negative control  Avian sarcoma virus CT10 oncogene homolog, domain #2  Neurite outgrowth factor or neutrophil cytosol factor 2 domain #1  Signal transducing adaptor molecule |
