# Supplementary material for: C3G forms complexes with Bcr-Abl and p38α MAPK at the focal adhesions in chronic myeloid leukemia cells: implication in the regulation of leukemic cell adhesion
Source: Cell Commun Signal. 2013 Jan 23;11:9. doi: 10.1186/1478-811X-11-9 (PMC3629710; doi:10.1186/1478-811X-11-9)
Supplement: Additional file 9: Table S2 — SH3 domain list Array II. [file 1478-811X-11-9-S9.doc]

| Additional Table 2. SH3 domain list Array II | | |
| --- | --- | --- |
| Position | Domain | Full name |
| A1, 2  B1, 2  C1, 2  D1, 2  A3, 4  B3, 4  C3, 4  D3, 4  A5, 6  B5, 6  C5, 6  D5, 6  A7, 8  B7, 8  C7, 8  D7, 8  A9, 10  B9, 10  C9, 10  D9, 10  A11, 12  B11, 12  C11, 12  D11, 12  A13, 14  B13, 14  C13, 14  D13, 14  A15, 16  B15, 16  C15, 16  D15, 16  A17, 18  B17, 18  C17, 18  D17, 18  A19, 20  B19, 20  C19, 20  D19, 20 | AbI2B  PI3a  GRAP-D1  VAV2-D1  GRB2L-D1  SP93  JIP1  VAV3-D1  Abl2  STAC  M3KA  VAV3-D2  CCBA  Tec  MY7A  VINE-D1  CRKL-D1  TRIP10  NCK2-D2  VINE-D3  CSKP  PIG2  NCK2-D3  c-Src  NCK1-D2  ARH6  RHG4  Control  NE-DLG  BCA1  SH31  ---  NOF2-D2  BIN1  SNX9  ---  OSF  EFS  UAS3  --- | Abl interactor protein 2  Phosphatidylinositol 3-kinase regulatory alpha subunit  GRB2-related adaptor protein, SH3 Domain #1  Vav-2 protein, SH3 Domain #1  Grb2-related adaptor protein 2, SH3 Domain #1  Channel associated protein of synapse-110  C-jun-amino-terminal kinase interacting protein 1  Vav-3 protein, SH3 Domain #1  Abelson-related protein; Arg  Stac protein  Mitogen-activated protein kinase kinase kinase 10  Vav-3 protein, SH3 Domain #2  Dihydropyridine-sensitive L-type, calcium channel beta-1 subunit  Tyrosine-protein kinase Tec  Myosin VIIa  Vinexin, SH3 Domain #1  CRK-like protein, SH3 Domain #1  Cdc42-interacting protein 4  Cytoplasmic protein NCK2, SH3 Domain #2  Vinexin, SH3 Domain #3  Peripheral plasma membrane protein CASK  1-phosphatidylinositol-4,5-bisphosphate phosphodiesterase gamma 2  Cytoplasmic protein NCK2, SH3 Domain #3  Cellular Rous Sarcoma viral oncogene homolog  Cytoplasmic protein NCK1, SH3 Domain #2  Rho guanine nucleotide exchange factor 6  Rho-GTPase-activating protein 4  Negative control  Presynaptic protein SAP102  CRK-associated substrate  SH3-containing GRB2-like protein 1  ---  Neutrophil cytosol factor 2, SH3 Domain #2  Myc box dependent interacting protein 1  Sorting nexin 9  ---  Osteoclast stimulating factor 1  Embryonal Fyn-associated substrate  UBASH3A  --- |
