# Supplementary material for: C3G forms complexes with Bcr-Abl and p38α MAPK at the focal adhesions in chronic myeloid leukemia cells: implication in the regulation of leukemic cell adhesion
Source: Cell Commun Signal. 2013 Jan 23;11:9. doi: 10.1186/1478-811X-11-9 (PMC3629710; doi:10.1186/1478-811X-11-9)
Supplement: Additional file 11: Method 2 — pET15b-NBKSXa vector cloning region. pET15b-NBKSXa cloning/expression region (modified from pET15b) indicating the histidine tag and the thrombin recognition sequence and cleavage site. [file 1478-811X-11-9-S11.doc]

**Additional method 2. pET15b-NBKSXa vector cloning region**

**
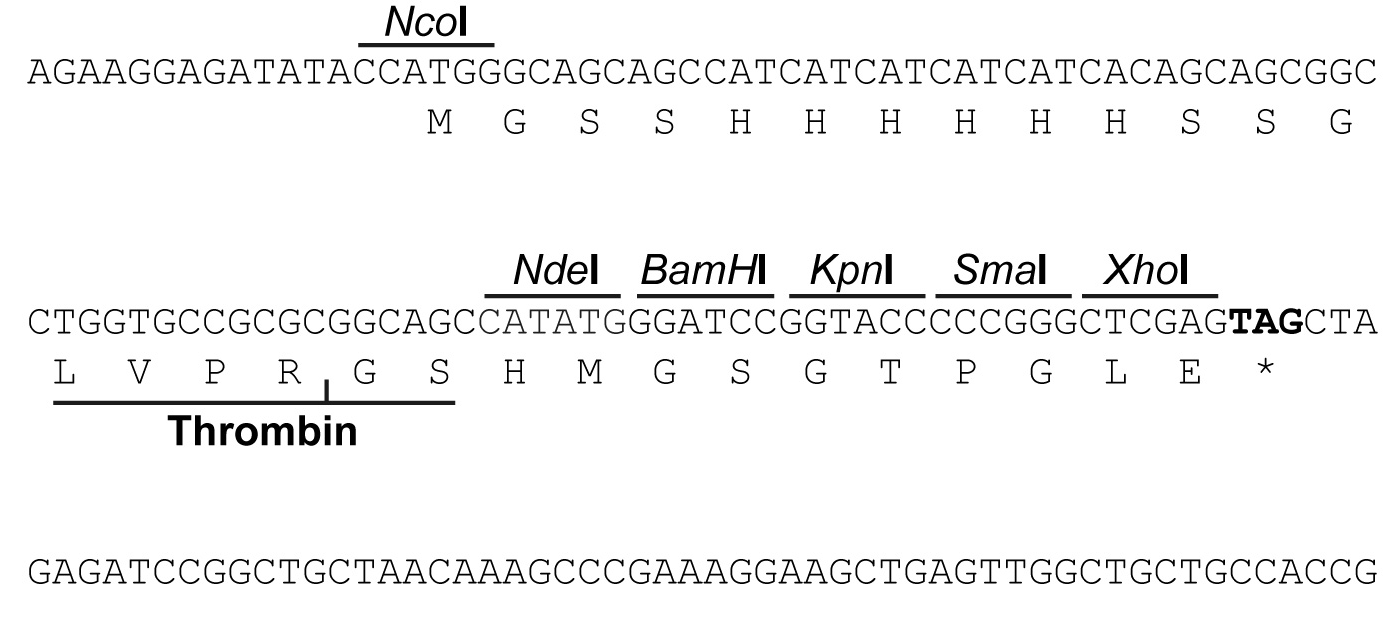
**

pET15b-NBKSXa cloning/expression region (modified from pET15b) indicating the histidine tag and the thrombin recognition sequence and cleavage site.
