# Supplementary material for: C3G forms complexes with Bcr-Abl and p38α MAPK at the focal adhesions in chronic myeloid leukemia cells: implication in the regulation of leukemic cell adhesion
Source: Cell Commun Signal. 2013 Jan 23;11:9. doi: 10.1186/1478-811X-11-9 (PMC3629710; doi:10.1186/1478-811X-11-9)
Supplement: Additional file 12: Method 3 — Cloning of C3G-SH3-b, Cbl-SH3-b or p130Cas-SH3-b domains into pETEV15b or pET15b-NBKSXa vectors. C3G-SH3-b domain was amplified by PCR with oligos C3GSH3-bF: 5´-GGGGAATTCCCATGGCTGGCATTCGGGTGGTTGAT-3´ and C3GSH3-bR: 5´-CCCGGATCCCTACTAACTGCCGTCTCTGCTGTCC-3´ and cloned into NcoI-BamHI sites of pETEV15b. Cbl-SH3-b domain was amplified with oligos CblSH3bBamHI-F:5´-GGGGGATCCCCGCCTTCTCCATTCTC-3´ and CblSH3bXhoI-R: 5´-CCCCTCGAGCTACTAAGGTGGCAGTTTTGGCAC-3´ and cloned into pET15b-NBKSXa by BamHI-XhoI digestion. p130CasP2-domain (proline-rich region 2) was amplified with oligos CasP2BamHI-F: 5´-AGGGGATCCTCACTGCTCTTCAGACGG-3 and CasP2XhoI-R: 5´-GGGCTCGAGCTACTAGGTGAACTTAGGGGGTGA-3´ and cloned into BamHI-XhoI sites of pET15b-NBKSXa. [file 1478-811X-11-9-S12.doc]

**Additional method 3. Cloning of C3G-SH3-b, Cbl-SH3-b or p130Cas-SH3-b domains into pETEV15b or pET15b-NBKSXa vectors.**

C3G-SH3-b domain was amplified by PCR with oligos C3GSH3-bF: 5´-GGGGAATTCCCATGGCTGGCATTCGGGTGGTTGAT-3´ and C3GSH3-bR: 5´-CCCGGATCCCTACTAACTGCCGTCTCTGCTGTCC-3´ and cloned into *Nco*I-*Bam*HI sites of pETEV15b. Cbl-SH3-b domain was amplified with oligos CblSH3bBamHI-F: 5´-GGGGGATCCCCGCCTTCTCCATTCTC-3´ and CblSH3bXhoI-R: 5´-CCCCTCGAGCTACTAAGGTGGCAGTTTTGGCAC-3´ and cloned into pET15b-NBKSXa by *Bam*HI-*Xho*I digestion. p130CasP2-domain (proline-rich region 2) was amplified with oligos CasP2BamHI-F: 5´-AGGGGATCCTCACTGCTCTTCAGACGG-3 and CasP2XhoI-R: 5´-GGGCTCGAGCTACTAGGTGAACTTAGGGGGTGA-3´ and cloned into *Bam*HI-*Xho*I sites of pET15b-NBKSXa.
